# Unraveling the Fundamental Mechanism of Interface Conductive Network Influence on the Fast-Charging Performance of SiO-Based Anode for Lithium-Ion Batteries

**DOI:** 10.1007/s40820-023-01267-3

**Published:** 2023-12-04

**Authors:** Ruirui Zhang, Zhexi Xiao, Zhenkang Lin, Xinghao Yan, Ziying He, Hairong Jiang, Zhou Yang, Xilai Jia, Fei Wei

**Affiliations:** 1 Beijing Key Laboratory of Green Chemical Reaction Engineering and Technology, Department of Chemical Engineering, Tsinghua University, Beijing, 100084 People’s Republic of China; 2 School of Materials Science and Engineering, University of Science and Technology Beijing, Beijing, 100083 People’s Republic of China; 3 Beijing Key Laboratory of Chemical Power Source and Green Catalysis, School of Chemistry and Chemical Engineering, Beijing Institute of Technology, Beijing, 100081 People’s Republic of China; 4 Institute of Polymer Science and Engineering, Department of Chemical Engineering, Tsinghua University, Beijing, 100084 People’s Republic of China

**Keywords:** Fast charging, SiO anode, Interface conductive network, Ionic transport, Mechanical stability

## Abstract

- Influence of interface conductive network on ionic transport and mechanical stability under fast charging is explored for the first time.
- The mitigation of interface polarization is precisely revealed by the combination of 2D modeling simulation and Cryo-TEM observation, which can be attributed to a higher fraction formation of conductive inorganic species in bilayer SEI, and primarily contributes to a linear decrease in ionic diffusion energy barrier.
- The improved stress dissipation presented by AFM and Raman shift is critical for the linear reduction in electrode residual stress and thickness swelling.

**Abstract:**

Progress in the fast charging of high-capacity silicon monoxide (SiO)-based anode is currently hindered by insufficient conductivity and notable volume expansion. The construction of an interface conductive network effectively addresses the aforementioned problems; however, the impact of its quality on lithium-ion transfer and structure durability is yet to be explored. Herein, the influence of an interface conductive network on ionic transport and mechanical stability under fast charging is explored for the first time. 2D modeling simulation and Cryo-transmission electron microscopy precisely reveal the mitigation of interface polarization owing to a higher fraction of conductive inorganic species formation in bilayer solid electrolyte interphase is mainly responsible for a linear decrease in ionic diffusion energy barrier. Furthermore, atomic force microscopy and Raman shift exhibit substantial stress dissipation generated by a complete conductive network, which is critical to the linear reduction of electrode residual stress. This study provides insights into the rational design of optimized interface SiO-based anodes with reinforced fast-charging performance.
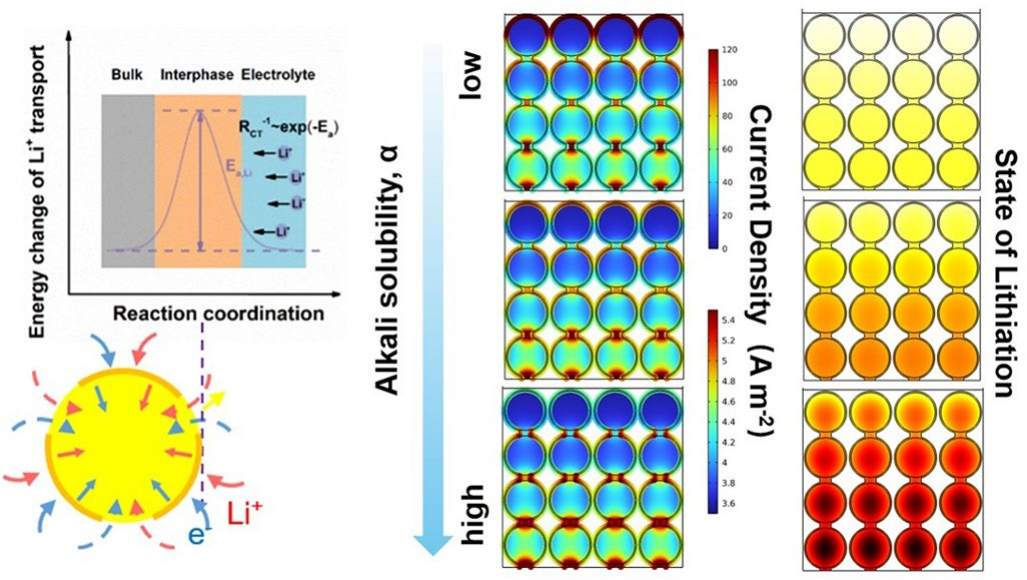

**Supplementary Information:**

The online version contains supplementary material available at 10.1007/s40820-023-01267-3.

## Introduction

To achieve carbon neutrality, efficiency obtained via electrification transportation has opened up the new era of lithium-ion batteries (LIBs) at a trend of blooming growth. Despite rapid progress in long mileage and stable economy, consumer acceptance and unsatisfied occupancy still pose a challenge for power and consumer markets such as electric vehicles (EV) and portable devices [1]. In addition to exploring a high-capacity electrode system, the promotion of fast-charging capability has gathered increasing attention with regard to addressing the posing barrier of “range anxiety” for potential new owners [2]. The electrode-related overpotential induced by high current densities during fast charging mainly governs the fast-charging property. The overpassing-limit overpotential results in interface side reactions and accelerates cell failure [3–5]. Charge transfer occurring in the solid electrolyte interphase (SEI) and cathode electrolyte interphase sides is one of the main limitations that hinder the fast-charging performance; the efficient reduce of the electrode/electrolyte interface kinetic barrier is regarded as the key challenge to realize the superb capability at a high rate [6, 7]. With regard to anodes, a commercial graphite anode possesses inferior kinetics and low working potential (approximately 0.1 V vs. Li/Li^+^); when charging over 1C, several issues, specifically lithium plating, cause severe capacity degradation and thermal runaway [8–11]. By contrast to graphite, the high theoretical capacity (~ 4200 mAh g^−1^) for silicon-based anode permits the reduction of electrode thickness without sacrificing the integral energy density, and the relatively high lithiation potential (~ 0.4 V) abates lithium plating-induced issues [12–14]. However, huge volume expansion (~ 400%) and inherent poor conductivity (*σ* ~ 10^−3^ S cm^−1^ and *D*_Li_^+^  ~ 10^−11^ cm^2^ s^−1^) significantly hamper the practical fast-charging capability [15–17]. SiO-based anodes possess better comprehensive electrochemical properties owing to moderate volume expansion (~ 120%) and nearly 1–2 orders of magnitude higher intrinsic conductivity (*σ* ~ 10^−1^ S cm^−1^, *D*_Li_^+^ ~ 10^−9^ cm^2^ s^−1^) compared to Si, which displays congenital advantages for fast charging [18–20]. However, they are still far away from large-scale applications, thereby demanding for further improvement in overall conductivity and stability. Among several existing approaches, the introduction of high-conductive agents to construct an enhanced conductivity interface has been widely implemented to address the aforementioned issues [5, 21, 22]. To boost the practical commercialization, graphene-like highly conductive carbon with low tortuosity is ideally required for deriving quick short ionic and electronic pathways to enhance transport efficiency. Meanwhile, the robust mechanical stability of an interface is essential for ensuring conductive network integrity to impede the loss of electrical contact, specifically during fast charging [23–26]. Highly conductive interface construction can improve the fast-charging property; however, its quality governs the overall performance. Nevertheless, so far, the underlying mechanism of how the quality of interface conductive network to influence the transport behavior, mechanical stability, and quantification relationship between the microstructures with respect to performance is yet to be systemically researched and understood.

Herein, the two main questions about SiO-based anode performance under high-rate conditions, the ionic transfer activation energy (*E*_a-Li_) and mechanical stability are focused on a deeper understanding of the correlation between the interface conductive network quality and overall performance. Three representative composites with different integrity requirements (represented as alkali solubility, *α*) are selected. First, dynamic nanoindentation with the compress–depress conductivity test is performed to depict the relationship between the mechanical properties and the integrity of the interface network. Second, the ionic transport activation energy obtained based on temperature-varied electrochemical impedance spectra (EIS) is evaluated. Based on the 2D modeling simulation and micro-morphology observation of SEI via Cryo-TEM, a mechanism with respect to the influence of the interface network quality on the ionic transport property is precisely confirmed. Finally, the mechanical stability after fast charging is analyzed based on the investigation of electrode thickness swelling and roughness variation, combined with the residual stress calculated by the Raman shift; the influence of the interface conductive network in structure durability is revealed. With the combined experimental and modeling efforts, a deeper understanding on underlying mechanism of performance reinforcement by regulating the interface conductive network is a great significance to develop high-performance batteries with preferable fast-charging properties.

## Experimental Section

### Materials

The commercial SiO powder (Flotu, ~ 3 μm) was first dispersed in a glucose solution with a 60% solid content under stirring for 1 h. Subsequently, the homogeneous suspension underwent a spraying–drying process to obtain secondary particles, and the whole process was conducted in a Φ50 fluidized bed reactor. After the system temperature reached 800 °C under a constant flow of argon to remove the air, the carbon source (C_2_H_4_) was introduced into the reactor at 2:3 (C_2_H_4_/Ar) in the total gas hourly space velocity of 300, 450, and 600 h^−1^, respectively. The system temperature was maintained for 30 min until the ethylene decomposition reaction was completed and the carbon was coated on the surface of SiO particles completely using CVD. When the system temperature was cooled down naturally to 25 °C, black powder was obtained.

### Reactions

A rectangular plastic bag (90 cm × 30 cm) was tailored for subsequent alkali dissolution reactions; 9 g of KOH was dissolved in 150 mL of deionized water to prepare the alkali solution after complete dissolution; 150 mg SiO@C powder sample was added to the bag while adding 30 g of the as-prepared KOH solution, which was followed by encapsulation without leakage. The encapsulated bag was placed under a water bath at a constant temperature of 60 °C, and the volume change of the bag was recorded using the drainage method during the reaction at the set time point.

### Characterizations

The morphology and structure were observed using a scanning electron microscope (JEOL Ltd., Tokyo, Japan) at 3 kV and Cs-corrected FEI Titan G2 60–300 kV transmission electron microscope (FEI Ltd., Hillsboro, USA) equipped with an elemental analyzer at 120 kV. The morphology of SEI under high resolution was recorded using a 2100 plus transmission electron microscope (JEOL Ltd., Japan) at 120 keV with an Elsa.698.STP cooling system. The crystal structure, chemical composition, and chemical bond of the materials were characterized based on X-ray diffraction (XRD) with an X-ray powder diffractometer (D8-Advance, Bruker, Germany) in a 2θ range of 5°–90°, XPS, EDS (ESCALAB 250Xi, America), and Raman spectra (LabRam HR800, Horiba Jobin–Yvon, France). Thermogravimetric analysis (TGA) with a TGA/DSC1 STARe system was performed at 30–800 °C and 10 °C min^−1^ in air. The surface chemical compositions of electrodes were identified based on the X-ray photoelectron microscopy （XPS） measurements (Thermo Fisher ESCALAB 250Xi, USA). The atomic force microscopy (AFM) system (Bruker Corp., Dimension Icon) was employed to collect the surface toughness evolution images of electrodes before and after cycling. 

### Measurements

The working electrodes were prepared via the mixture of active materials, Super P as the conductive agent, carboxymethyl cellulose (CMC) as the thickening agent, and polymerized styrene butadiene rubber (SBR) as the binder to form a slurry in a weight ratio of 80:10:5:5. The aqueous slurry was coated on a Cu foil using an automatic thick film coater (MSK-AFA-ES200) with mass loading on the electrode of 2–2.8 mg cm^−2^. The separator was a Celgard 2500 membrane, and the lithium foil was employed as a counter electrode. The electrolyte was 1 M LiPF_6_ in an ethylene carbonate (EC)/diethyl carbonate (DEC)/dimethyl carbonate (DMC) mixture with a volume ratio of 1:1:1, and 10 wt% fluoroethylene carbonate (FEC) was the additive agent. The 2032-type coin cells were assembled in an argon-filled glove box (O_2_ < 0.1 ppm; H_2_O < 0.1 ppm). The electrochemical performance test was evaluated based on galvanostatic charge–discharge protocols in a NEWARE CT-4008Tn multi-channel battery test system at room temperature. For half cells, the Si-based electrodes were cycled between 0.01 and 1.5 V (vs. Li/Li^+^). Cyclic  voltammogram (CV) test and electrochemical impedance spectroscopy (EIS) were performed using the VSP-300 potentiostat system (BioLogic Science Instruments Ltd.). EIS was performed at 100 kHz to 0.1 Hz.

### Modeling

A 2D model was employed to simulate the lithiation of the SiO@C anodes, where a high current density of 5 mA cm^−2^ was applied. During modeling, the current distribution, mass transport, and charge transfer were coupled. Two modules in COMSOL, lithium-ion battery and diluted species transmission, were used to solve the time-dependent equations. The Li-ion insertion kinetic was used to model charge transfer on active material particles. The equilibrium potential of Li in active materials was obtained from the experimental data of coin cells at a low lithiation rate of 0.1 A g^−1^, which was then normalized as a function of the state of lithiation ([Li^+^]/[Li^+^]_max_) (Fig. S21). The counter electrode was defined as lithium metal. A cutoff voltage of 0.01 V was applied as the stop condition of lithiation. While modeling these three materials with various alkali solubility, the conductivity of particles (*σ*_s_), exchange current density (*i*^0^), and diffusion coefficient (*D*_s_) in the solid phase were set up according to the experimental results; other parameters were all the same, including the diameter of the particle, the diffusion coefficient of the electrolyte and the porosity of the separator. Model descriptions and parameters are listed in Tables S1 and S2.

### Theories

For fast charging, overpotential (∆*φ*_CT_) is the main driving force to govern the charge transfer of lithium at the interface between the electrode and electrolyte. To simplify the explanation, the direct contact between the electrode material and electrolyte with no additional interfacial layers hinders charge transfer, and the overpotential is determined based on lithium-ion transfer instead of electrons that are assumed. The overpotential can be decided using the classical Butler–Volmer equation, which is related to the interfacial current *I* [27–29]:

1 $$I = j_{0} A\left[ {\exp \left( {\frac{\alpha F}{{RT}}\Delta \varphi_{{{\text{ct}}}} } \right) - \exp \left( { - \frac{{\left( {1 - \alpha } \right)F}}{RT}\Delta \varphi_{{{\text{ct}}}} } \right)} \right]$$

here $j_{0}$ denotes the exchange current density, $A$ denotes the electrode area, $\alpha$ denotes the charge transfer coefficient that describes the potential landscape at the interface, $F$ denotes the Faraday constant, and $\Delta \varphi_{{{\text{ct}}}}$ denotes the overpotential. Further, $j_{0}$ can be expressed as:

2 $$j_{0} = j_{0}^{{{\prime } }} \exp \left( { - \frac{{E_{{\text{a}}} }}{RT}} \right)$$

$j_{0}^{{{\prime } }}$ can be obtained as a simplified expression for the exchange current density. Here, $j_{0}^{\prime}$ denotes a prefactor that is dependent on the concentrations of lithium ions, vacancies, and electrons in an active material and electrolyte, and $E_{{\text{a}}}$ denotes the energy barrier for lithium-ion diffusion. Therefore, the charge transfer resistance ($R_{{{\text{ct}}}}$) can be obtained through the following equation [30]:

3 $$\frac{1}{{R_{{{\text{ct}}}} }} = \frac{{z{FA}j_{0}^{{{\prime } }} }}{{{RT}}}\exp \left( { - \frac{{E_{{\text{a}}} }}{{{RT}}}} \right)$$

## Results and Discussion

### Theoretical and Structural Characterizations

**Fig. 1:**
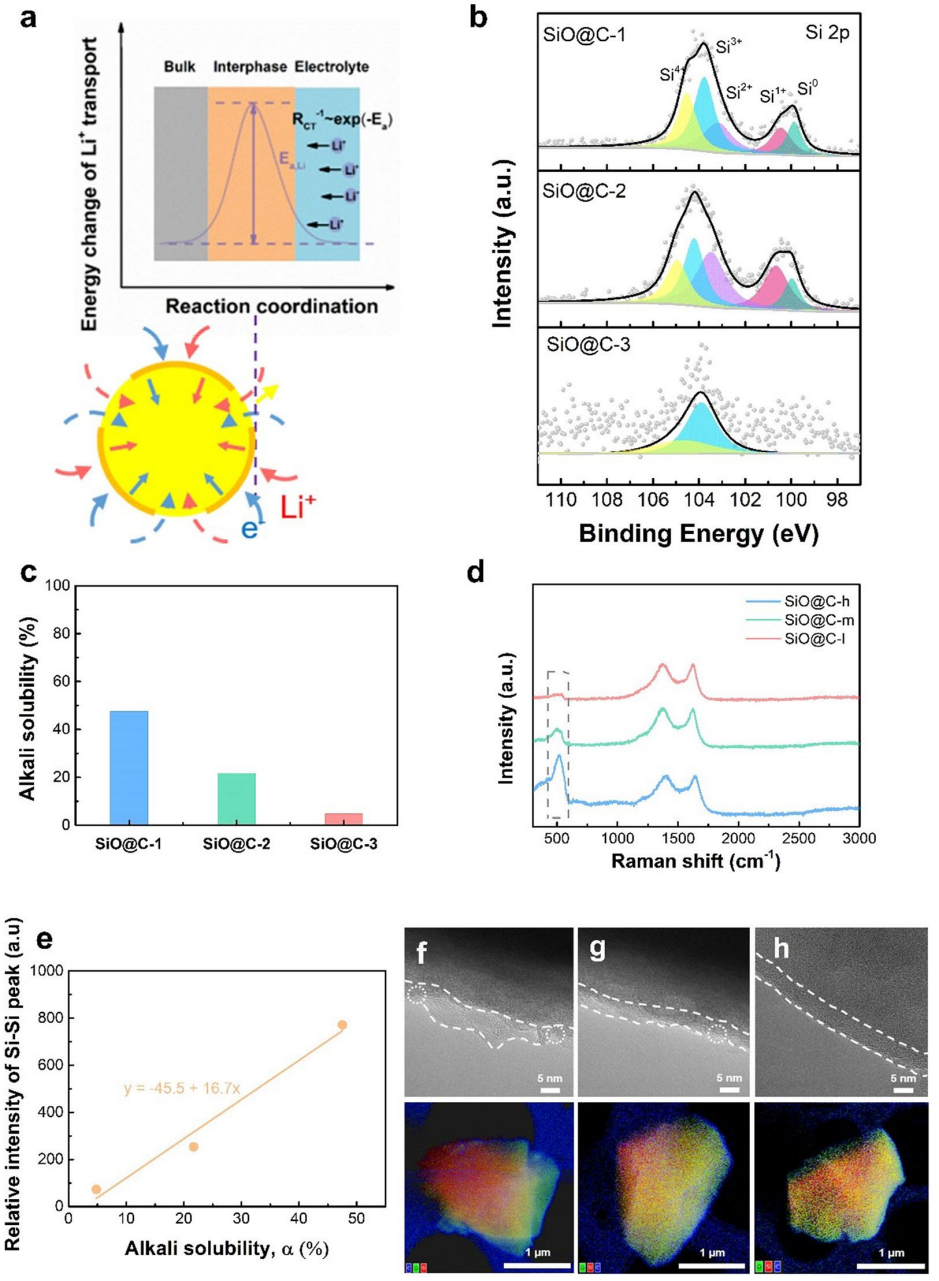
Theoretical, structural, and physical analysis of three representative SiO-based composites. **a** Li^+^ diffusion at the interface based on the BV equation. **b** High-resolution Si 2*p* XPS spectra. **c** Alkali solubility, **d** Raman spectra, and **e** the relationship between the relative intensity of the characteristic Si peak with alkali solubility for three representative SiO-based composites with different interface networks (varying quality). High-resolution TEM images with the corresponding EDS images of **f** SiO@C-h, **g** SiO@C-m, and **h** SiO@C-l

According to the aforementioned theoretical basics, reducing the diffusion activation energy ($E_{{\text{a}}}$) is the most direct and effective method to effectively decrease the charge transfer resistance *R*_ct_ at the interface to promote fast charging (Fig. 1a). For surface modification, it is reasonable to speculate that high-conductivity interface construction with better integrity can more effectively reduce the activation energy and improve the isotropy of high-efficiency ion transport throughout the interface. However, the specific impact on ion transport behavior and its underlying mechanism are yet to be understood. To investigate the influence of the interface network on electrochemical performance, three representative SiO-based composites were prepared via fluidized bed high-temperature CVD under three different fluidization conditions with similar carbon content, ~ 4.8% (Fig. S5).

The three representative SiO-based composites were denoted as SiO@C-1, SiO@C-2, SiO@C-3, corresponding to the total gas hourly space velocity of 300, 450, and 600 h^−1^ (Sect. 2.1). The structural analysis was firstly performed using XPS. In the high-resolution spectra of Si 2*p* (Fig. 1b), the raw profile can be deconvoluted into five individual peaks, which correspond to Si (0) (99.1 eV), Si (+ 1) (100.6 eV), Si (+ 2) (103.6 eV), Si (+ 3) (104.0 eV), and Si (+ 4) (104.8 eV) [31–33]. The gradual decrease in the Si (0) signal reveals the improved coverage of the graphite-like carbon interface network to more effectively suppress the elemental Si exposure. Using the previously developed method to test coating integrity, the alkali solubility (α) of the three representative composites is quantified, 47.5%, 21.7%, and 4.8%, respectively (Fig. 1c). Therefore, in the following discussion, the three representative composites are redefined as SiO@C-h, SiO@C-m, and SiO@C-l, which represent the SiO@C composites with high, medium, and low alkali solubility, respectively. Furthermore, the two characteristic peaks appeared at 1338 and 1584 cm^−1^ in the Raman spectra for the three representative composites; they are attributed to the D and G bands of carbon. The *I*_D_/*I*_G_ ratio is approximately 0.8 for all the composites, demonstrating graphite-like carbon coating construction with superb conductivity [34–36]. With regard to the Si characteristic peak at 520 cm^−1^, considering the almost same coating thickness, the difference in intensity is evident; the lower intensity implies the enhanced coverage effect is provided by the graphite-like carbon interface network (Fig. 1d). Significantly, the relative intensity of the characteristic Si peak at ~520  cm^−1^ linearly increases with alkali solubility (Fig. 1e), indicating that the considerable difference in inner active Si exposure for the three composites can be reflected from micro-characterization and macro-inspection evaluation. The high-resolution TEM and the corresponding energy-dispersive X-ray spectroscopy (EDS) images, as shown in Fig. 1f–h, indicate that the SiO-based composites with different α values exhibit distinct variations in surface morphology. The composite with a low α value displays flat interface conductive network construction; by contrast, the uneven surface with more defects can be observed for the composite with a high α value. The aforementioned results depict the notable component and morphology differences of the three representative composites.

**Fig. 2:**
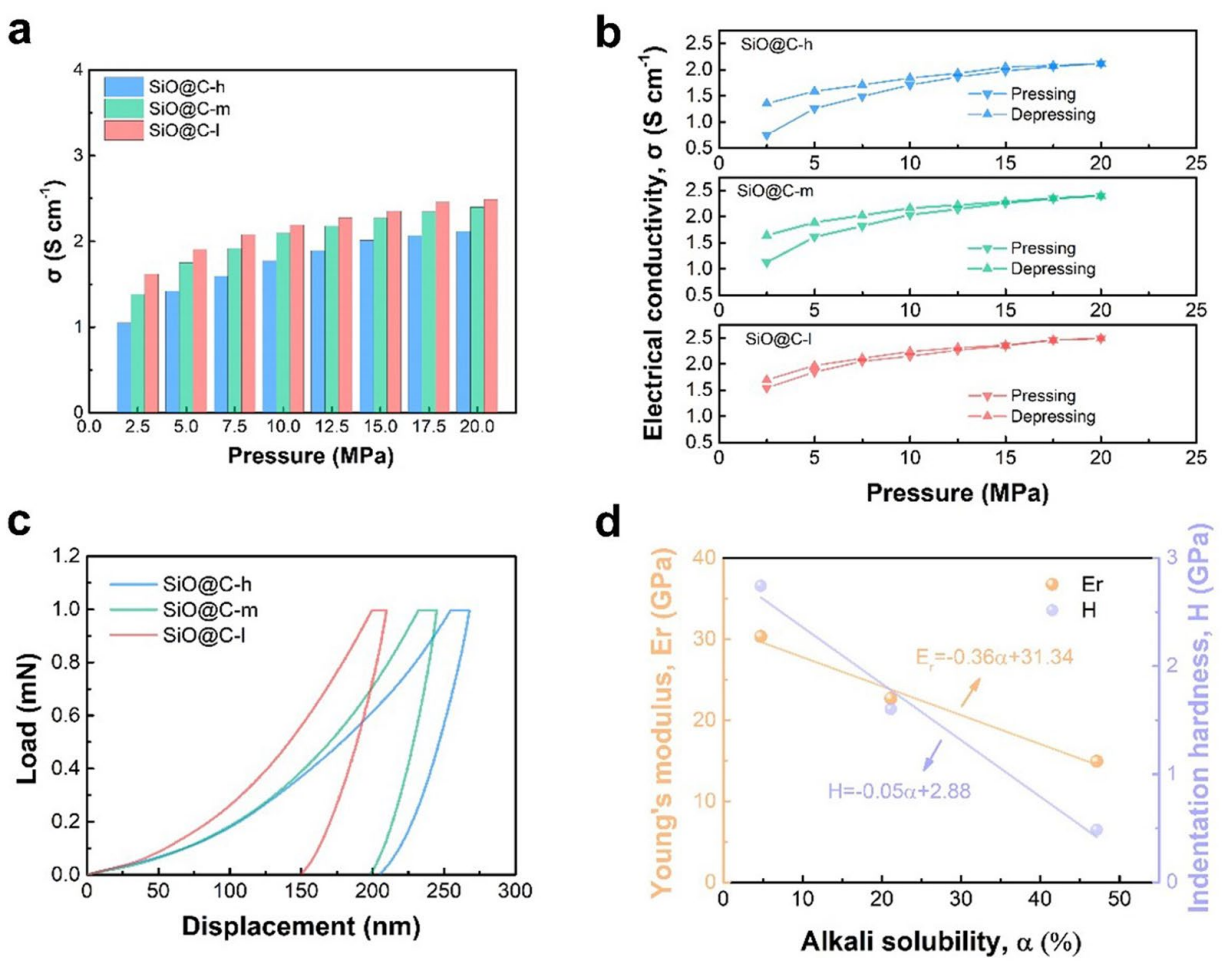
Electrical conductivity and mechanical stability investigation. **a** Average electrical conductivity under varying pressure for the composite anodes with varying alkali solubility. **b** Electrical conductivity changes with pressure during the pressing–depressing process. **c** Load–displacement curves of the composite anodes with varying alkali solubility. **d** Relationship between stiffness and hardness considering alkali solubility

The powder electrical conductivity test and related mechanical property characterizations are performed to further investigate the integral electrical conductivity and mechanical properties of the composites. The average conductivity under different pressures can be obtained from the pressing and depressing processes (Fig. 2a). The SiO@C-l composite exhibits the highest conductivity at different pressure levels, which reveals that the higher integrated interface conductive network improves the overall conductivity. Furthermore, the conductivity changes in the pressing–depressing process are recorded to investigate the mechanical reversibility resisting the pressure. The notable difference, specifically in the low-pressure region, which corresponds to the variable of compact density induced by irreversibility after pressing, reflects the mechanical endurance for different composites (Fig. 2b). Intriguingly, with the improved quality of the interface network, the smaller gap of conductivity obtained during the pressing–depressing process confirms the improved mechanical reversibility to resist the structural damage caused by the external load. The nanoindentation test that evaluated the mechanical property of the three representative composites reveals that under the same load, the displacement of the composite with low α reduces, indicating that the structural deformation caused by stress can be efficiently reduced (Fig. 2c). The aforementioned compression test indicates that fracture toughening is achieved through enhanced interface network integrity to dissipate high mechanical strain accompanying the phase transition, specifically at a high charging rate. Moreover, the relationship between the critical mechanical parameters, Young’s modulus and indentation hardness, and the quality of the interface conductivity network is shown in Fig. 2d. Young’s modulus and the indentation hardness linearly decrease with an increase in *α*, indicating the influence of the quality on stiffness and hardness. The enhanced mechanical property of an interface conductive network can more effectively resist the strain triggered in the fast-charging lithiation process to maintain integral structure stability.

### Electrochemical Performance

**Fig. 3:**
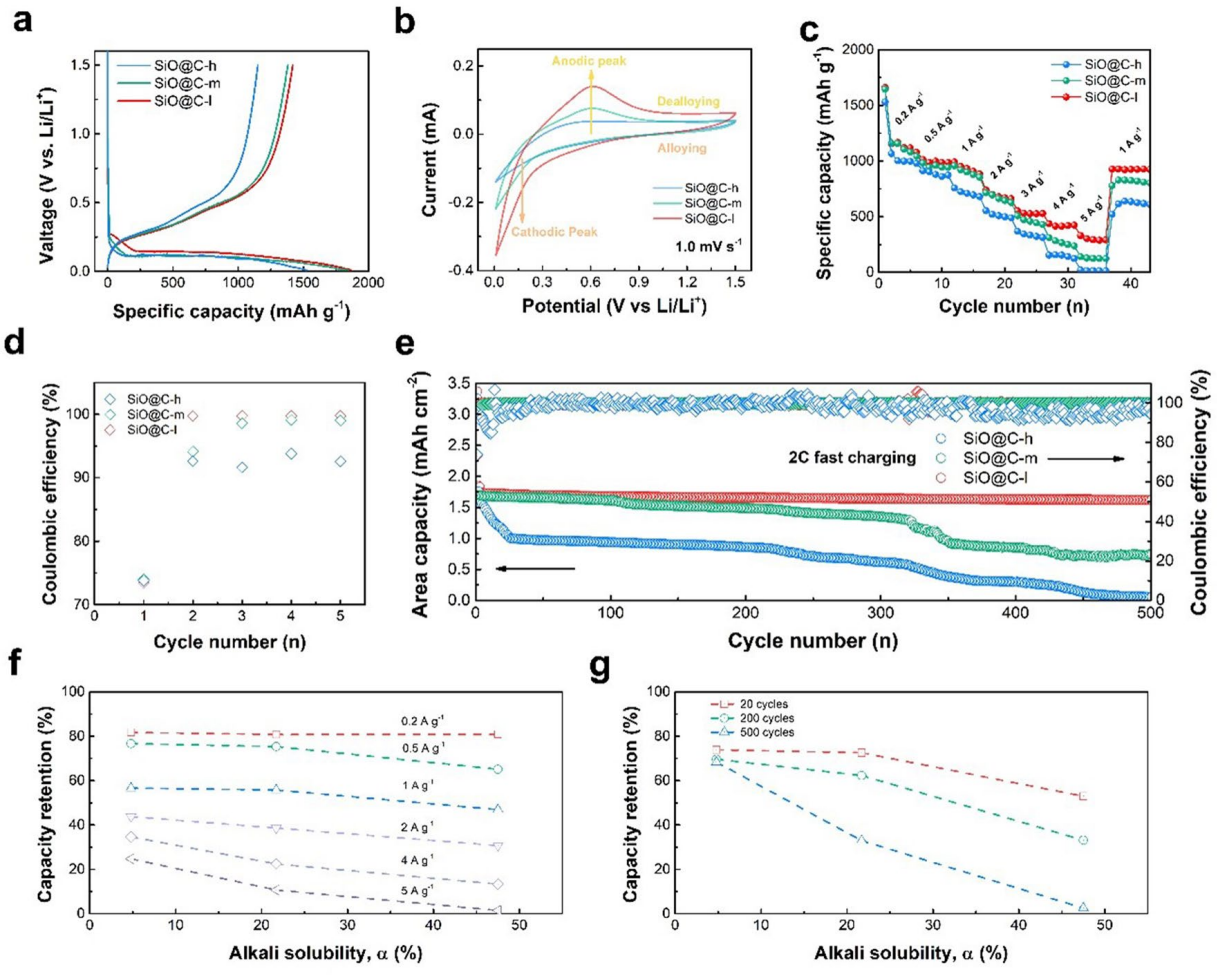
Electrochemical performance of alkali solubility composites. **a** Initial capacity–voltage profiles. **b** Initial CV curves at a scan rate of 1.0 mV s^−1^. **c** Rate cycling performance. **d** Coulombic efficiency changes within five cycles. **e** Cycling performance at 0.01–1.5 V and 2C. Capacity retention versus alkali solubility considering **f** different current intensities and **g** long-term cycling at 2C

The influence of interface network quality on electrochemical performance is systematically studied in Fig. 3. The first cycle of the capacity–voltage profiles is presented in Fig. 3a, which demonstrates the kinetics property of electrode. The relative high lithiation voltage for the composite with the lowest α value indicates mitigated polarization and improved Li-ion diffusion kinetics behavior displays a higher specific discharge capacity of 1865 mAh g^−1^ [37–39]. Furthermore, the rate performance and corresponding capacity retention are further analyzed. The cyclic voltammograms (CV) profile of the electrodes with different composites is exhibited at 1.0 mV s^−1^ (Fig. 3b). The weak cathodic peak at approximately 0.2 V is related to the alloying process of Li_*x*_Si [40–42]. The broad anodic peak appearing at approximately 0.5 V is ascribed to the dealloying processes of Li_x_Si and a part of lithium silicate oxide, Li_2_Si_2_O_5_ [43, 44]. The composite with the lowest α value displays higher response current compared to those of other electrodes, indicating the strengthened electrochemical activity owing to the promoted electrode activation degree. At 0.2–5 A g^−1^, the composites with the lowest *α* values delivers 1217, 994, 933, 689, 533, 422, and 301 mAh g^−1^, which are superior to those of the other two composites (Fig. 3c). Moreover, the long cycling stability at 2C is further considered to display the difference under the fast-charging condition. A higher initial coulombic efficiency (ICE) of 74.5% and a rapid increase in CE (> 99% after two cycles) are observed (Fig. 3d); the average CE within the first five cycles is 94.5%, which is higher than that of SiO@C-m (93.0%) and SiO@C-h (88.9%), implying a stable SEI and higher activation degree of the electrode. At cutoff voltages of 0.01–1.5 V, the composite with the lowest α value exhibits enhanced stability; after the initial cycle, the area capacity is consistent at a high level of approximately 1.5 mAh cm^−2^. Nearly 90% of the capacity is retained within 500 cycles, higher than that of 44.8% for the composite with the moderate α value, and the composite with the highest *α* value cannot exhibit reversible capacity (Fig. 3e). Importantly, the capacity retention of the composite with the lowest α value possesses an advantage under the high current intensity (Fig. 3f). The aforementioned capacity retention of 20% for the composite with the lowest α value is achieved at 5 A g^−1^, which is remarkably higher than those of the other two composites. When the current density was recovered till the middle level of 1 A g^−1^, a reversible capacity of 99.2% is conserved; in contrast, 90.1% and 85.4% are maintained for the other two composites, demonstrating the superb reversibility for the composite with the lowest α value under the high-rate discharge–charge condition. At a fast-charging rate of 2C, the difference in the capacity loss is evident within the prolonged cycles (Fig. 3g); the capacity retention of the composite with the highest α value has decreased to < 40% after 200 cycles. In contrast, the composite with the lowest α value enhances stability with a minimal loss during the 500 cycles. Excellent cyclability under fast charging can further verify the enhanced interface conductivity quality and facilitate higher stability to effectively restrain the prominent volume effect for maintaining the integral structure stability and protecting the electrical contact from delamination from each other; therefore, high stable area capacity can be acquired.

### Kinetics Evaluation and Modeling Effort

**Fig. 4:**
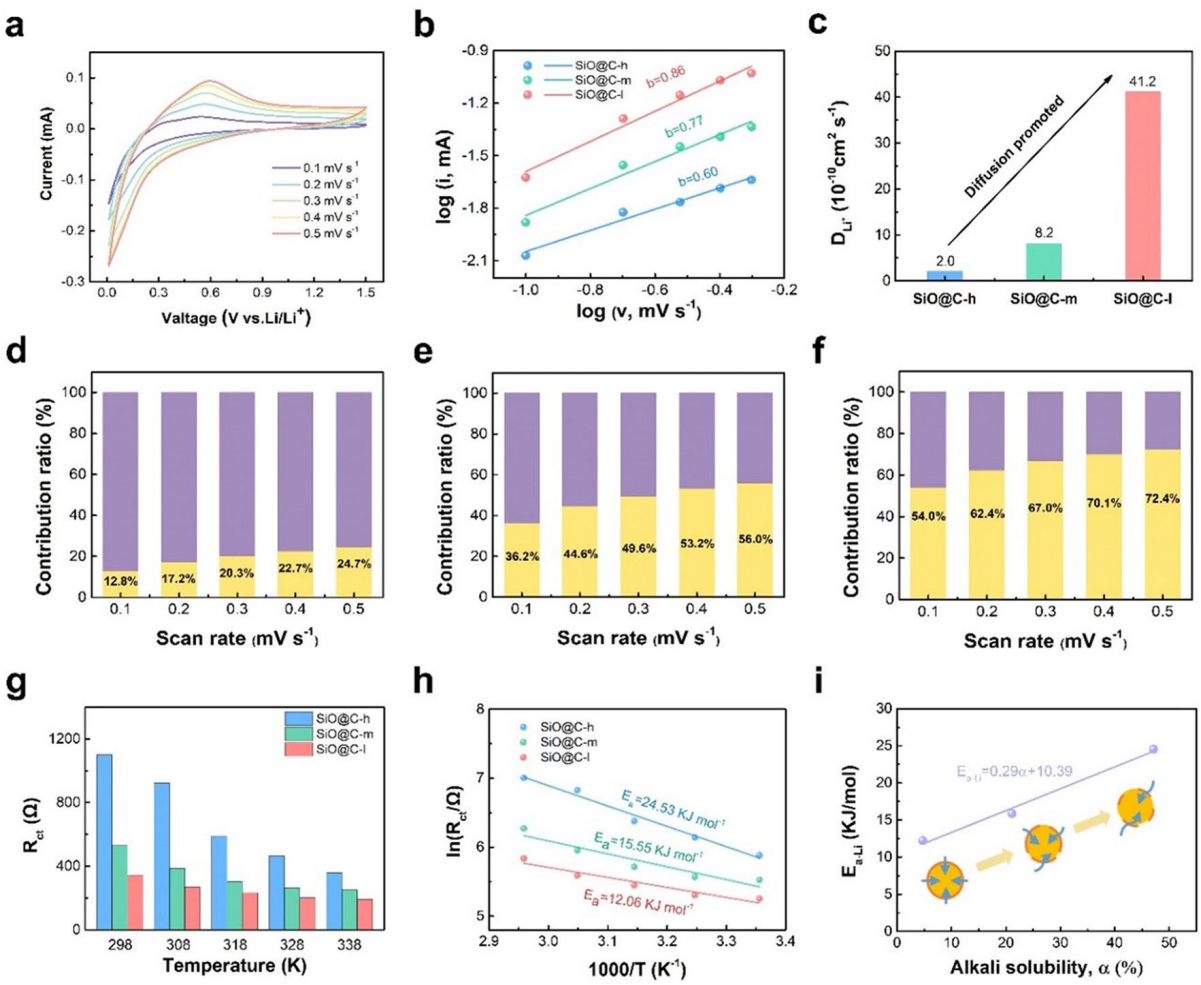
Electrode kinetics analysis. **a** CV curves at 0.1–0.5 mV s^−1^ for SiO@C-l electrode. **b** Fitted straight line of *I*_p_ versus scan rate and corresponding. **c** *D*_Li_^+ ^of as-prepared SiO@C-h, SiO@C-m, SiO@C-l electrodes. **d**–**f** Ratio of capacitance-controlled and surface-controlled capacity of the as-prepared SiO@C-h, SiO@C-m, SiO@C-l electrodes. **g** Temperature variable (*R*_ct_) of different alkali solubility composite anodes. **h** Linear fitting of reciprocal *R*_ct_ versus reciprocal temperature and *E*_a_ of Li^+^ diffusion through the interface. **i** Linear fitting of *E*_a-Li_ with alkali solubility

To further explore the kinetic mechanism for investigating the lithium-ion diffusion behavior, CV tests at different scan rates were performed (Figs. 4a and S9–S10). With respect to the high linearity between the responding current and scan rate, the electrode kinetics can be identified as a surface-controlled rate-limiting process. Meanwhile, the linear relationship between the current and the square root of the scan rate can be identified as a diffusion-controlled rate-limiting process [44, 45]. Therefore, the total electrochemical capacity can be divided into two parts, surface and diffusion contributions, which can be determined using the following equation:

4 $$i = av^{b}$$

where *i* denotes the responding current, *v* denotes the scan rate, and *a* and *b* denote the parameters related to the electrode behavior. *b* = 0.5 represents the diffusion-dominated kinetics, and *b* = 1.0 represents the classical kinetics controlled using the surface-dominated process [46–48]. The *b* values are 0.60, 0.77, and 0.86 for SiO@C-h, SiO@C-m, and SiO@C-l, respectively, which are obtained through fitting as shown in Fig. 4b. Furthermore, the apparent Li^+^ diffusion coefficient (*D*_Li_^+^) can be obtained by applying the Randles–Sevcik equation [49, 50]:

5 $$I_{{\text{P}}} = 2.69 \, \times \, 10^{5} n^{3/2} {AD}_{{{\text{Li}}}}^{1/2} v^{1/2} C_{{\text{o}}}$$

where *I*_P_ denotes the corresponding peak current, *n* denotes the number of electrons participating in the reaction, *A* denotes the electrode surface area, *D*_Li_^+^ denotes the Li-ion apparent diffusion coefficient, *v* denotes the scan rate, and *C*_o_ denotes the Li^+^ concentration in the electrolyte. The *D*_Li_^+^ values of SiO@C-h, SiO@C-m, and SiO@C-l are 2.04 × 10^−10^, 8.15 × 10^−10^, and 4.12 × 10^−9^ cm^2^ s^−1^, respectively, as shown in Fig. 4c. The value that is nearly 20 times the *D*_Li_^+^ value of SiO@C-l can be attributed to the improved interface network with higher integrity, which can help derive more highly efficient pathways facilitating the ionic transfer. In addition, the contribution ratio with regard to surface and diffusion can be determined using the following equation:

6 $$i = k_{1} v + k_{2} v^{1/2}$$

where *k*_*1*_*v* and* k*_*2*_*v*^1/2^ denote the contribution ratios related to surface and diffusion to the total responding current, respectively. As the scan rate increases, the capacitive contribution ratio reaches 72.4% at 0.5 mV s^−1^ for SiO@C-l. In contrast, the contribution ratios of SiO@C-h and SiO@C-m are 24.7% and 56.0%, respectively, indicating that the surface-controlled kinetics become more dominated for the composite with the improved interface conductive network (Fig. 4d, e). EIS at different temperatures was conducted to explore the Li-ion diffusion activation energy (*E*_a-Li_) at the interface (Figs. S11–S13) [51]. The obtained *R*_ct_ values at different temperatures are shown in Fig. 4g; among all the electrodes, SiO@C-l exhibits the minimum *R*_ct_ value, indicating the lowest Li^+^ transfer resistance at the interface. The relationship between *R*_ct_ and 1/*T* can reflect the Li-ion diffusion energy barrier at the interface. The *E*_a-Li_ value of SiO@C-l (12.06 kJ mol^−1^) is lower than those of SiO@C-h (24.53 kJ mol^−1^) and SiO@C-m (15.55 kJ mol^−1^) (Fig. 4h), representing the lowest Li^+^ migration resistance caused by the improved interface conductive network construction. Importantly, the Li-ion diffusion energy barrier reveals a close correlation with the quality of the interface conductive network; the linear increase in the alkali solubility indicates the importance of improving the integrity of an interface conductive network, which can indeed decrease the ionic diffusion energy barrier to enhance transport efficiency for improving the fast-charging performance (Fig. 4i). With the low-*α* composite, the high-quality integrated interface network can help provide highly efficient pathways all over the surface; the overall ionic diffusion resistance on the interface can be extensively reduced to achieve rapid transfer. However, a low-quality interface network results in a higher anisotropy of ion conduction at the interface, thereby increasing the integral diffusion resistance to limit fast charging.

**Fig. 5:**
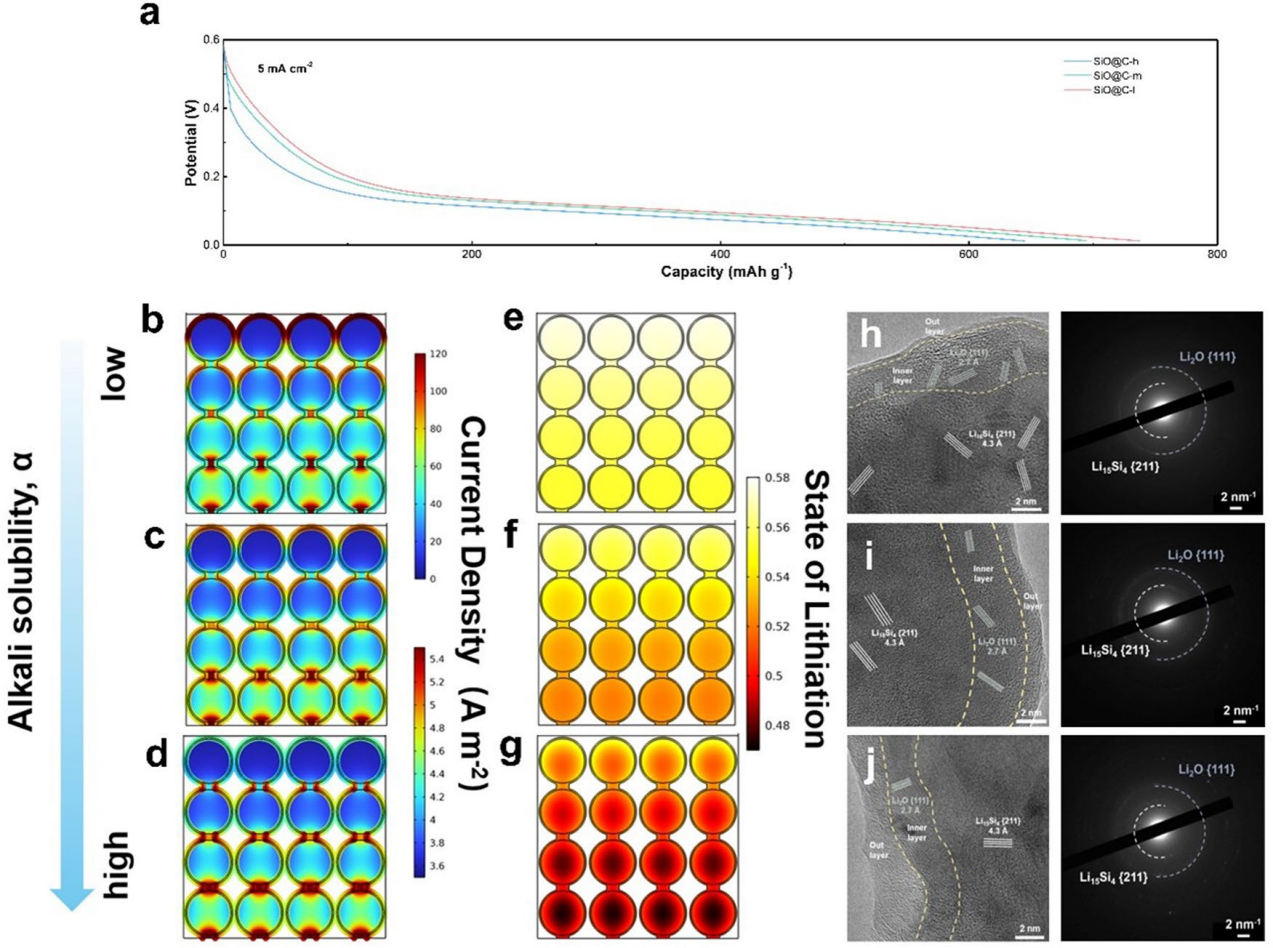
2D modeling results based on the experimental results and interface morphology observation. **a** Simulated discharge curves for different alkali solubility composite anodes. The 2D modeling results at the end of discharge at 5 mA cm^−2^ are displayed.  **b**–**d** The current density in the solid phase.** e**–**g** The state of lithiation reported for different alkali solubility composite anodes. **h**–**j** Cryo-TEM images and SAED patterns of SiO@C-l, SiO@C-m and SiO@C-h

To gain further insights into the underlying mechanism of an interface network with respect to performance improvement, a 2D model was developed based on the experimental results and considered as an input for simulation. The three conditions at a high discharge current level (5 mA cm^−2^) are compared (Fig. 5a). Additional details on the modeling endeavor, e.g., model description and relation parameter description, are provided (model description and parameters, Supporting Information). Based on the simulated discharge curve at 5 mA cm^−2^, SiO@C-l exhibits a specific capacity of 737.2 mAh g^−1^, which is higher than those of SiO@C-m (693.9 mAh g^−1^) and SiO@C-h (644.6 mAh g^−1^). This variation can be attributed to a higher overpotential for the composite with a lower quality interface conductive network, which results in a shorter discharge time before reaching the cutoff voltage of 0.01 V. An average discharge voltage gap of approximately 0.06 V between the SiO@C-l and SiO@C-h is observed. To investigate the origin of this overpotential, the distributions of the interface and bulk current density for particles at the end of the lithiation state are plotted in Fig. 5b–d. For SiO@C-l, the actual particles near the electrolyte exhibit a higher interface current density of 5.5 A m^−2^ than those of SiO@C-m (4.8 A m^−2^) and SiO@C-h (4.2 A m^−2^). The aforementioned results indicating the improved integrity of the interface network enhance the interface current intensity by facilitating the charge transfer process. However, the inferior interface network with high-alkali-solubility composites exhibits a more sluggish diffusion behavior, higher resistance at the interface results in a higher voltage drop, resulting in a sooner arrival at the end of discharge cutoff potential, which is detrimental to the fast-charging performance.

Furthermore, the differences stemming from the integrity of the interface network for various particles are demonstrated in detail via lithiation ([Li]_SiO@C_/[Li]_SiO@C,max_) (Figs. 5e–g and S17–S21). A more uniform and higher state of lithiation (SOL) for the SiO@C-l electrode implies a better utilization of the active material particles, whereas a notable difference in SOL has occurred in the part near the electrolyte and bulk for the SiO@C-m electrode; this trend is exacerbated for the SiO@C-h electrode, which reveals non-uniformity and a higher fraction of low-SOL particles. The particles with higher SOL concentrate near the electrolyte instead of the current collector. The SOL gradient is the characteristic of a system limited by ionic diffusion, considering that the particles far from the electrolyte (the source of the ions) possess lower SOL. Specifically, fast charge transfer leads to low activation polarization, resulting in the domination of diffusion in an electrolyte, whereas sluggish diffusion in a solid phase and retarded charge transfer attributed to an inferior interface network make ionic diffusion in an electrolyte less important; however, charge transport in a solid phase dominates. Moreover, the non-existence of an SOL gradient in the bulk phase of particles implies that solid-state diffusion in the bulk phase may not limit the electrochemical reaction with an improved interface network even at a high lithiation rate. The modeling process relying on the experimental results further verifies the influence of a higher integrated interface network for enhancing charge transfer efficiency at the interface to decrease the resistance. Although the model does not exhibit the long-term benefits obtained from the improved interface network, it still possesses merits at a high rate. Moreover, the improved kinetics is supported by the galvanostatic intermittent titration technique (GITT) [52–55], in which the *D*_Li_^+^ values of SiO@C-l are relatively higher than that of other two composites at most of the SOLs (Fig. S30). The microstructure based on the SEI observation can further confirm the influence of the interface network quality on interface chemistry. The formed SEI at the end of discharge was characterized via Cryo-TEM, which can provide the original morphology and structure information with no damage for SEI investigation [56–58]. The Cryo-TEM SEI images and corresponding selected area electron diffraction (SAED) are obtained. For the lithiated state, the SEI reveals a dual-layer structure; the inner layer comprises inorganic compounds, including crystalline Li_2_O, and the outer layer comprises amorphous organic species, which jointly construct SEI with a bilayer structure (Fig. 5h–j). For SiO@C-l, a higher fraction of the Li_2_O crystal phase can be identified in the inner layer. Meanwhile, the thickness of the amorphous layer is approximately 1 nm, and the layer exhibits uniform and thin characteristics. As the alkali solubility increases, the occurrence of Li_2_O decreases in the inner layer; moreover, the thicker and inhomogeneous amorphous layer is formed on the surface. In addition, the higher emergence of Li_2_CO_3_ can be confirmed when the electrode is in the delithiated condition (Fig. S23). The high ionic conductivity of the two inorganic species generated via Li_2_O and Li_2_CO_3_ can enhance the mechanical stability and diffusivity of the interface to further reduce the volume change and improve the overall transfer efficiency. The enhanced interface conductivity network maintains the integrity stability itself and helps promote interface stability. Under fast charging condition, a more robust SEI bilayer with better comprehensive properties can be achieved in the thinner amorphous outer layer owing to the restriction of electrolyte decomposition, and the formation of inorganic species can jointly make contribution to the reduction of interface diffusion resistance.

### Mechanical Stability

**Fig. 6:**
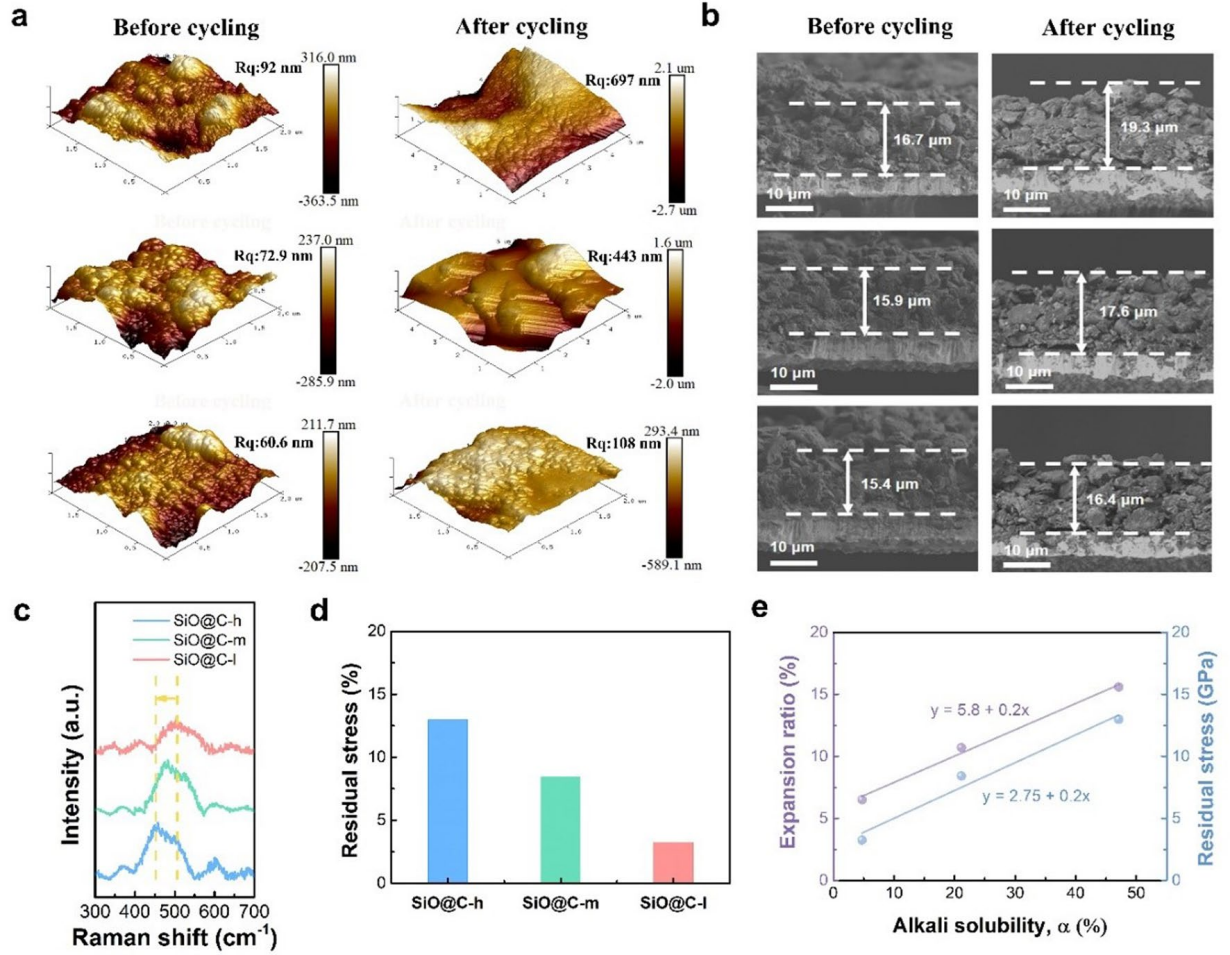
Mechanical stability analysis after cycling. **a** AFM images of the SiO@C-h, SiO@C-m, and SiO@C-l (α decreases from up to down) electrodes before and after fast-charging cycling. **b** Cross-sectional SEM images of the SiO@C-h, SiO@C-m, and SiO@C-l (*α* decreases from up to down) electrodes before and after cycling. **c** Raman spectra at 250–750 cm^−1^ after cycling. **d** Residual stress of SiO@C-h, SiO@C-m, and SiO@C-l after cycling. **e** Relationship between the alkali solubility and electrode expansion ratio, as well as residual stress

The mechanical stability influenced by the interface conductivity network is further demonstrated after cycling. To further disclose the morphology evolution and thickness swelling of the electrode during fast charging, post-cycling half cells were disassembled and monitored. AFM measurements were used to investigate electrode surface roughness evolution during cycling. According to the 3D surface morphology of anodes (Fig. 6a), before cycling, the surface roughness (*R*q) values of SiO@C-h, SiO@C-m, and SiO@C-l were 92, 72.9, and 60.6 nm, respectively. After cycling, the 3D surface roughness value of SiO@C-l increased slightly to 108 nm. Conversely, the surface roughness values of SiO@C-m and SiO@C-h increased to 443 and 697 nm, respectively. Furthermore, the smaller change in the surface roughness reconfirmed that a higher quality interface conductive network can stabilize the electrode structures of SiO-based anodes by relieving huge stress, specifically under the fast-charging condition. The overall structural collapse caused by notable volume expansion is believed to be highly related to the existing stress during the repeated lithiation/delithiation process. The thickness swelling is precisely observed based on the cross-sectional SEM images. After cycling, the thickness of the high-alkali-solubility composite electrode increases from 16.7 to 19.3 μm, corresponding to an expansion rate of 15.6%. The moderately low-alkali-solubility composite electrodes exhibit lower expansion rates of 10.7% and 6.5%, implying that the volume change is efficiently suppressed (Fig. 6b). Moreover, the magnified SEM images indicate that after cycling, the SiO@C-l composite electrode surface is intact with slight cracks, whereas the SiO@C-m composite electrode exhibits cracks with thick SEI generation, and severe Si pulverization occurs and an invisible particle boundary is formed for the SiO@C-h composite electrode, indicating inferior structure stability under the fast-charging condition (Fig. S24). The smaller thickness swelling with slighter pulverized active particle shedding of the SiO@C-l composite electrode reveals that a higher quality interface conductive network can more efficiently buffer volume expansion and stabilize the electrode structure/interface, thereby facilitating the rapid kinetic behavior of SiO-based anodes under fast charging.

The post-cycling Raman spectra are displayed in Fig. 6c. The electrode with the high-alkali-solubility composite reveals the redshift of the Si characteristic peak, which corresponds to the existing residual stress after undergoing electrochemical reactions. The residual stress can be calculated using the following equation:

7 $$\sigma\, =\,\frac{{\left( {\omega - \omega_{0} } \right) \cdot 2\omega_{0} }}{{\left( {C_{11} + 2C_{12} } \right) \cdot \left( {p + 2q} \right)}} = 0.209\Delta \omega$$

where $\sigma$ denotes the residual stress remained in the electrode (GPa), $C_{11}$ = 76.8 × 10^−13^ m^2^ N^−1^ and $C_{12}$ =  − 21.4 × 10^−13^ m^2^ N^−1^ for Si, $p$ =  − 1.43 × 10^28^ s^−2^, $q$ =  − 1.89 × 10^28^ s^−2^, and $\Delta \omega$ denotes the shift in the wavenumber (cm^−1^) [59–62]. The residual stress values of the electrodes with different alkali solubility composites are calculated to be 3.25, 9.46, and 12.97 GPa (Fig. 6d). The effect of the higher residual stress induces severe structural degradation, which is reflected in the thickness change of the electrodes. The residual stress obtained from the Raman shift and electrode expansion ratio reveals a linear increase in alkali solubility (Fig. 6e). Insufficient coating integrity can lead to huge residual stress accumulation during prolonged cycling, specifically at high rates, which would ultimately lead to the failure of the overall structure. In contrast, as alkali solubility decreases, corresponding to the enhanced coating integrity, the improved elastic recovery provided by a complete conductive network contributes to improved stress dissipation, which is critical for a linear reduction in electrode residual stress. The suppression of integral electrode thickness variation and the better maintenance of electrical contact with the conductive network can help improve the fast-charging performance.

## Conclusions

In this work, the influence of the quality of an interface conductive network on the ionic transfer property and mechanical stability during fast charging were systematically studied considering three representative composites with different integrity requirements, which were represented by alkali solubility, α. The ionic transport activation energy (*E*_a-Li_) linearly increased with α, which indicates the importance of improving the interface network quality to promote fast-charging performance. The 2D modeling simulation results revealed an improved integrity of the interface network can enhance the interface current intensity to facilitate charge transfer and suppress interface polarization; this phenomenon can be ascribed to the construction of a more robust SEI bilayer, in which a higher fraction of the Li_2_O inorganic inner layer and the outer layer of thin amorphous organic species are presented. The improved stress dissipation generated by a complete conductive network was presented in detail via AFM and the Raman shift, which is critical for the linear reduction in electrode residual stress and thickness swelling to maintain the structural integrity, specifically during fast charging. The first-order reduction in ion diffusion activation energy and residual stress with alkali solubility α decreasing is probably the core of improving the quality of the interface conductive network for alloy-type SiO-based anodes to promote fast-charging performance. A higher ICE of 74.5% and then a rapid increase to > 99% after two cycles, an average CE of 94.5% within the first five cycles, and a capacity retention of nearly 90% within 500 cycles under 2C fast charging were achieved for the SiO-based composite with the lowest alkali solubility of 4.8%. This study deepens the fundamental mechanism understanding of the interface transport property and provides new insights into the rational design of high-capacity SiO-based anodes with an optimized interface to enhance fast-charging performance.

## Supplementary Information

Below is the link to the electronic supplementary material.

Supplementary file1 (DOCX 8987 kb)
