# Supplementary material for: Unraveling the Fundamental Mechanism of Interface Conductive Network Influence on the Fast-Charging Performance of SiO-Based Anode for Lithium-Ion Batteries
Source: Nanomicro Lett. 2023 Dec 4;16:43. doi: 10.1007/s40820-023-01267-3 (PMC10695911; doi:10.1007/s40820-023-01267-3)
Supplement: Supplementary file 1 — Supplementary file1 (DOCX 8987 kb) [file 40820_2023_1267_MOESM1_ESM.docx]

Supplemental File

**Unraveling the fundamental mechanism of interface conductive network influence on the fast-charging performance of SiO-based anode for lithium-ion batteries**

Ruirui Zhang^1,2,#^, Zhexi Xiao^1,#,^*, Zhenkang Lin^1,3^, Xinghao Yan^4^, Ziying He^1^, Hairong Jiang^1^, Zhou Yang^2^, Xilai Jia^2^, Fei Wei^1,^*

^1^ Beijing Key Laboratory of Green Chemical Reaction Engineering and Technology, Department of Chemical Engineering, Tsinghua University, Beijing, 100084, PR China

^2^ School of Materials Science and Engineering, University of Science and Technology Beijing, Beijing, 100083, PR China

^3^ Beijing Key Laboratory of Chemical Power Source and Green Catalysis, School of Chemistry and Chemical Engineering, Beijing Institute of Technology, Beijing, 100081, PR China

^4^ Institute of Polymer Science and Engineering, Department of Chemical Engineering, Tsinghua University, 100084, PR China

^#^ Ruirui Zhang and Zhexi Xiao contributed equally to this work.

* Corresponding author. E-mail: xzx14@tsinghua.org.cn, wf-dce@tsinghua.edu.cn


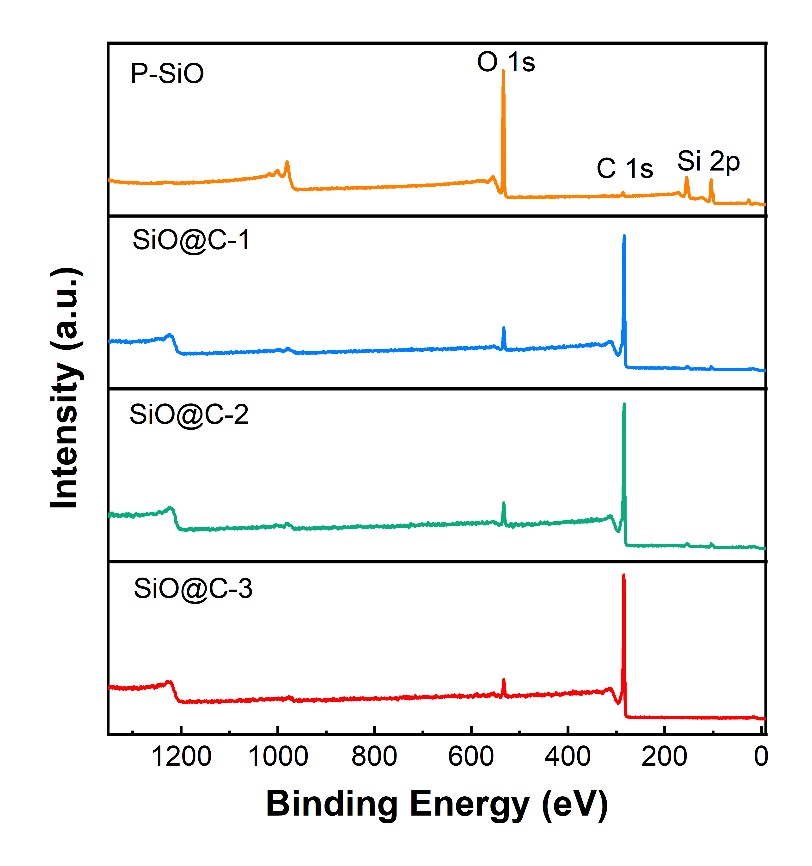


**Figure S1.** XPS overall spectra of different SiO@C composites.


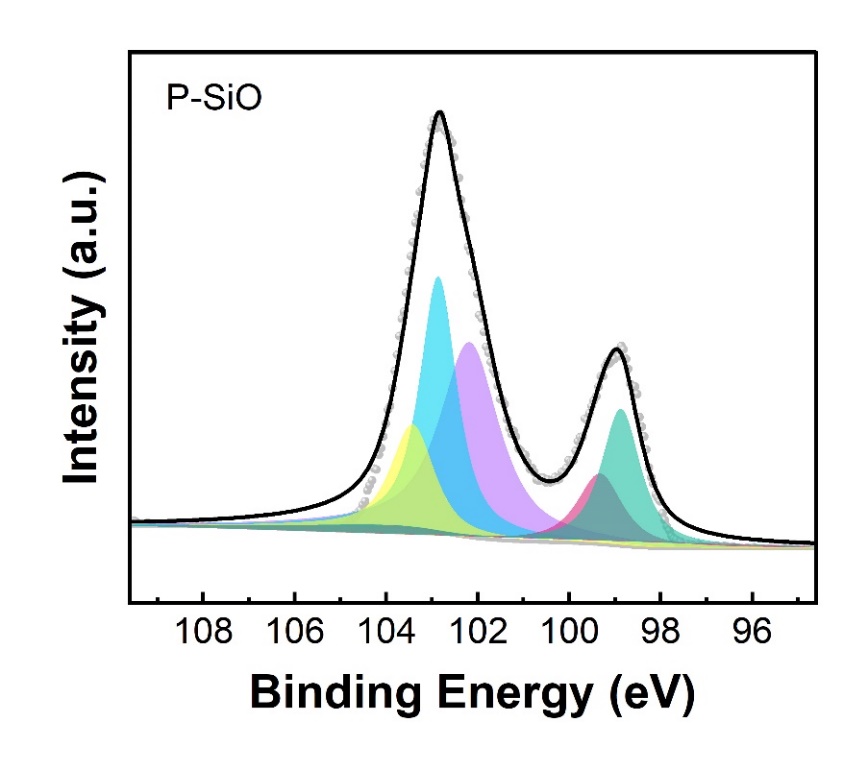


**Figure S2.** Si 2p XPS spectra of pristine SiO.





**Figure S3.** XRD patterns of SiO@C composites with different conductive network before cycling.





**Figure S4.** Dissolution rate change as the reaction time for different composites in 333 K.





**Figure S5.** The TG curves for different SiO@C composites.


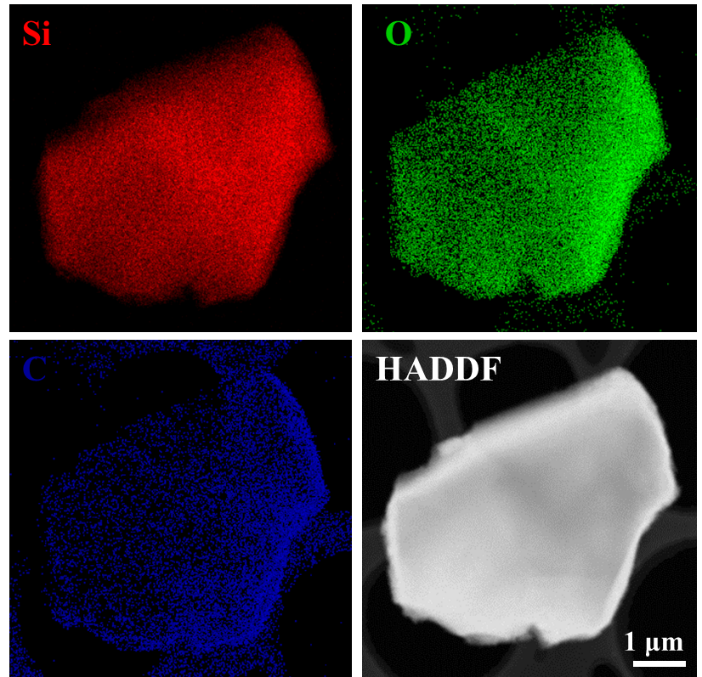


**Figure S6.** STEM and corresponding EDS images of SiO@C-l composite.


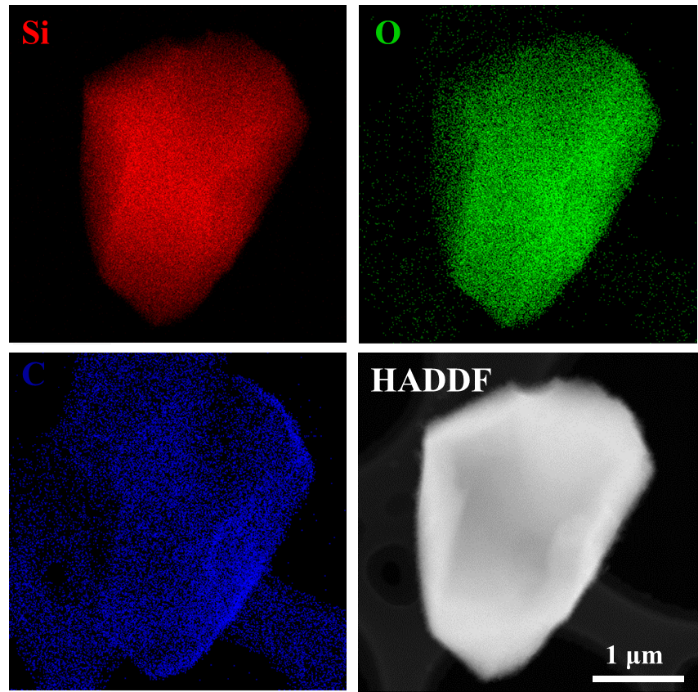


**Figure S7.** STEM and corresponding EDS images of SiO_x_@C-m composite.


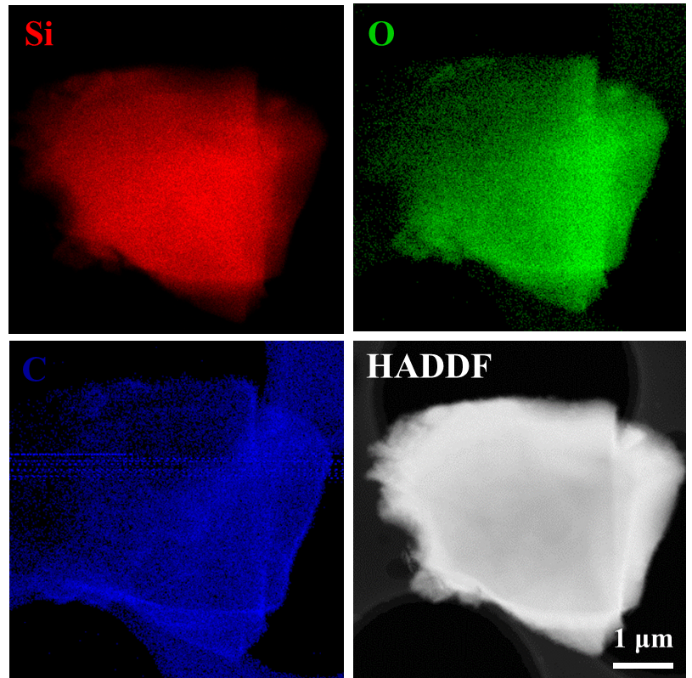


**Figure S8.** STEM and corresponding EDS images of SiO_x_@C-h composite.





**Figure S9.** CV curves of SiO@C-m composite from 0.1~0.5 mV s^-1^.





**Figure S10.** CV curves of SiO@C-h composite from 0.1~0.5 mV s^-1^.


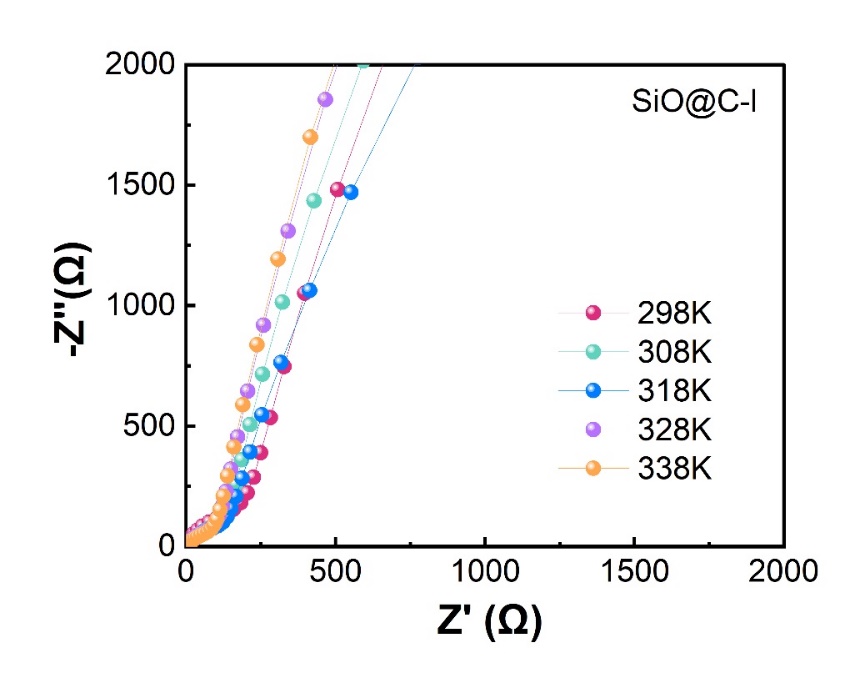


**Figure S11.** EIS spectra of SiO@C-l composite under different temperature.


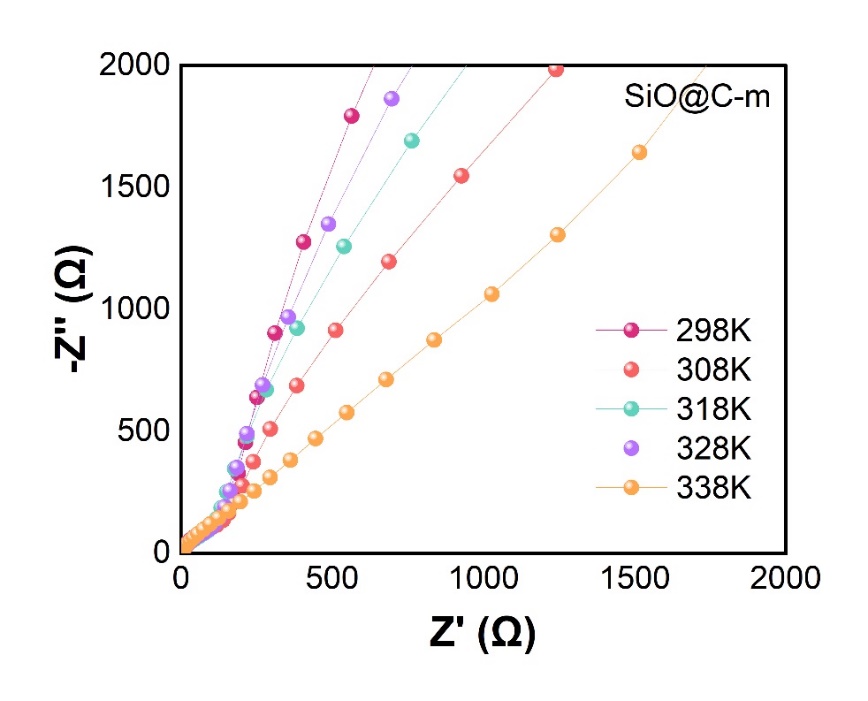


**Figure S12.** EIS spectra of SiO@C-m composite under different temperature.


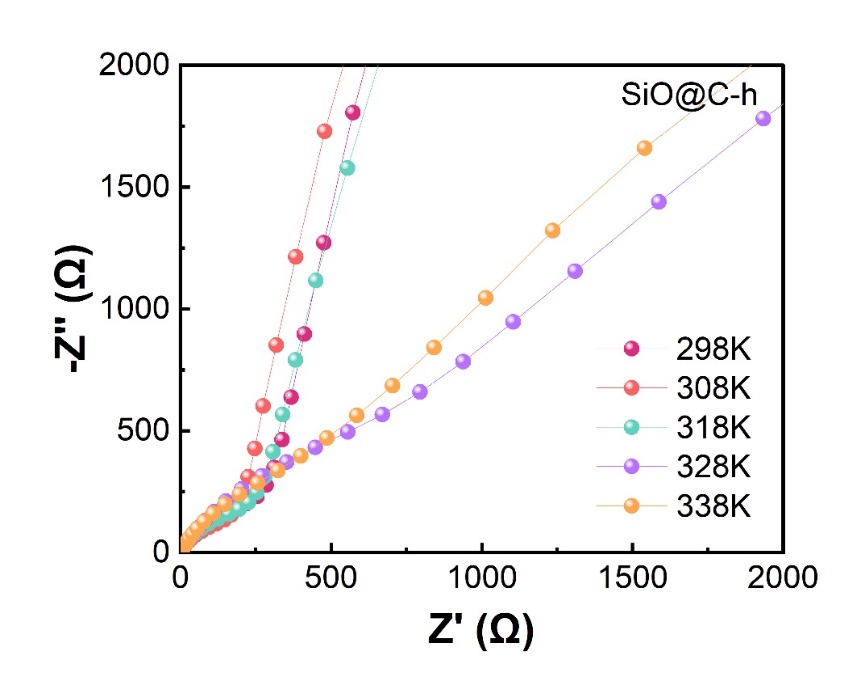


**Figure S13.** EIS spectra of SiO@C-h composite under different temperature.


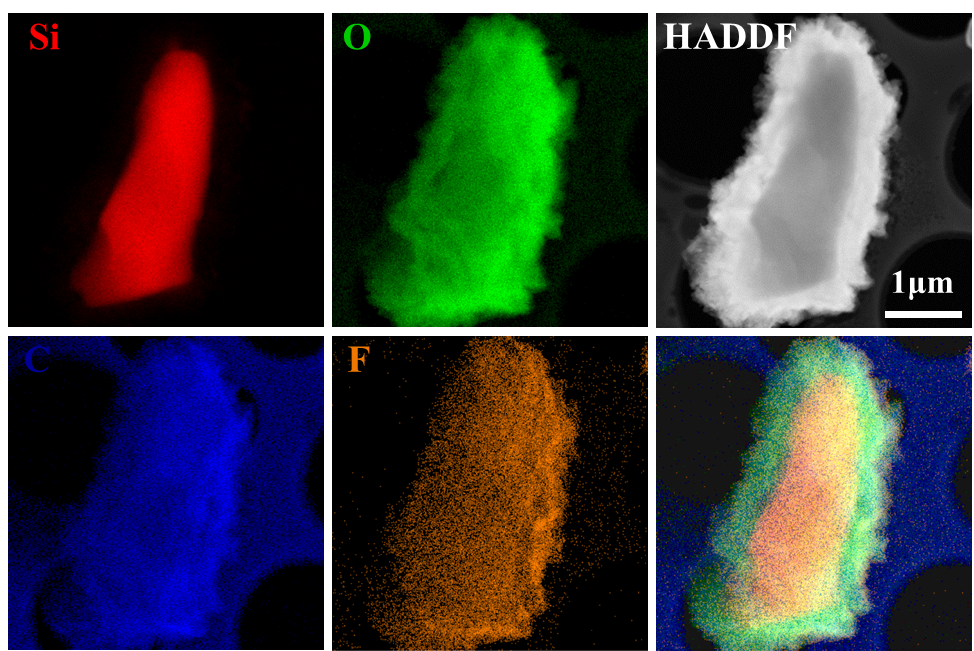


**Figure S14.** STEM and corresponding EDS images of SiO_x_@C-1 composite after cycling.


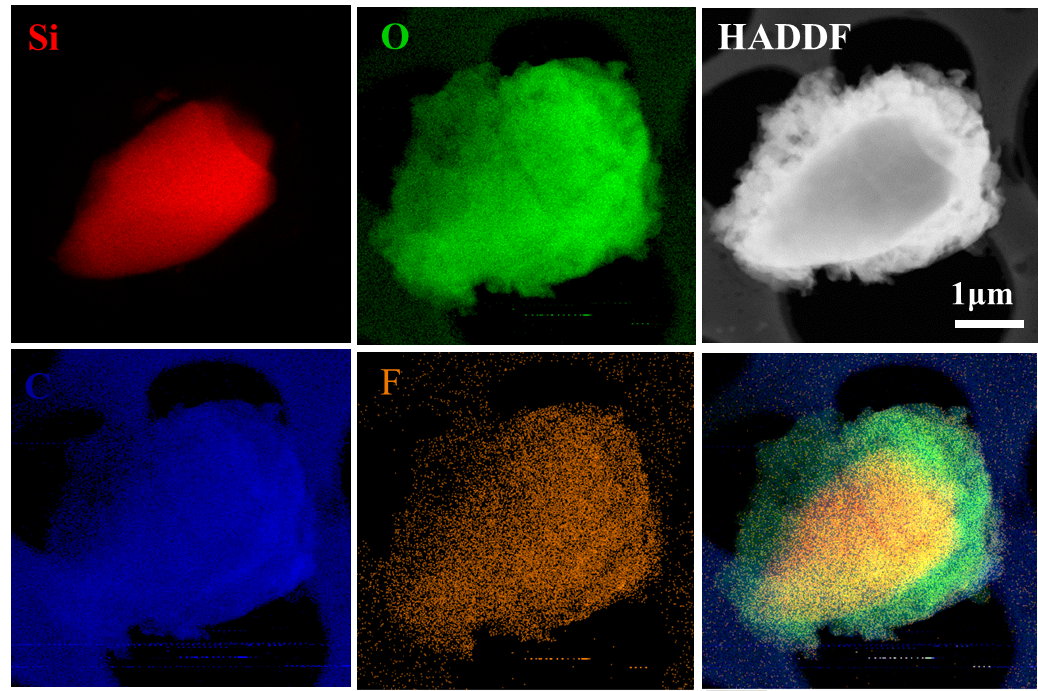


**Figure S15.** STEM and corresponding EDS images of SiO_x_@C-m composite after cycling.


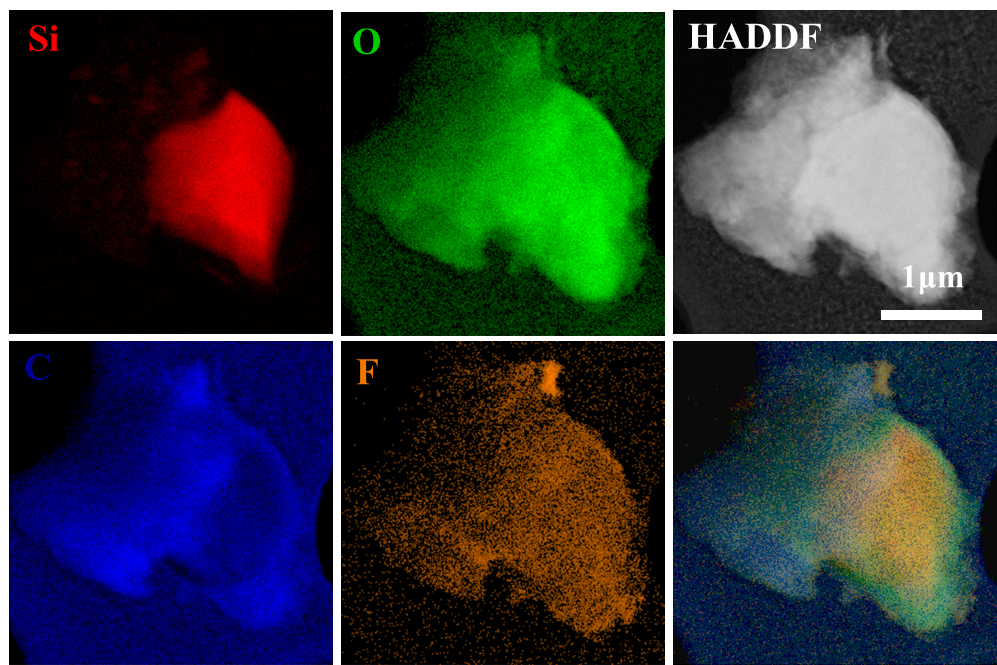


**Figure S16.** STEM and corresponding EDS images of SiO_x_@C-h composite after cycling.


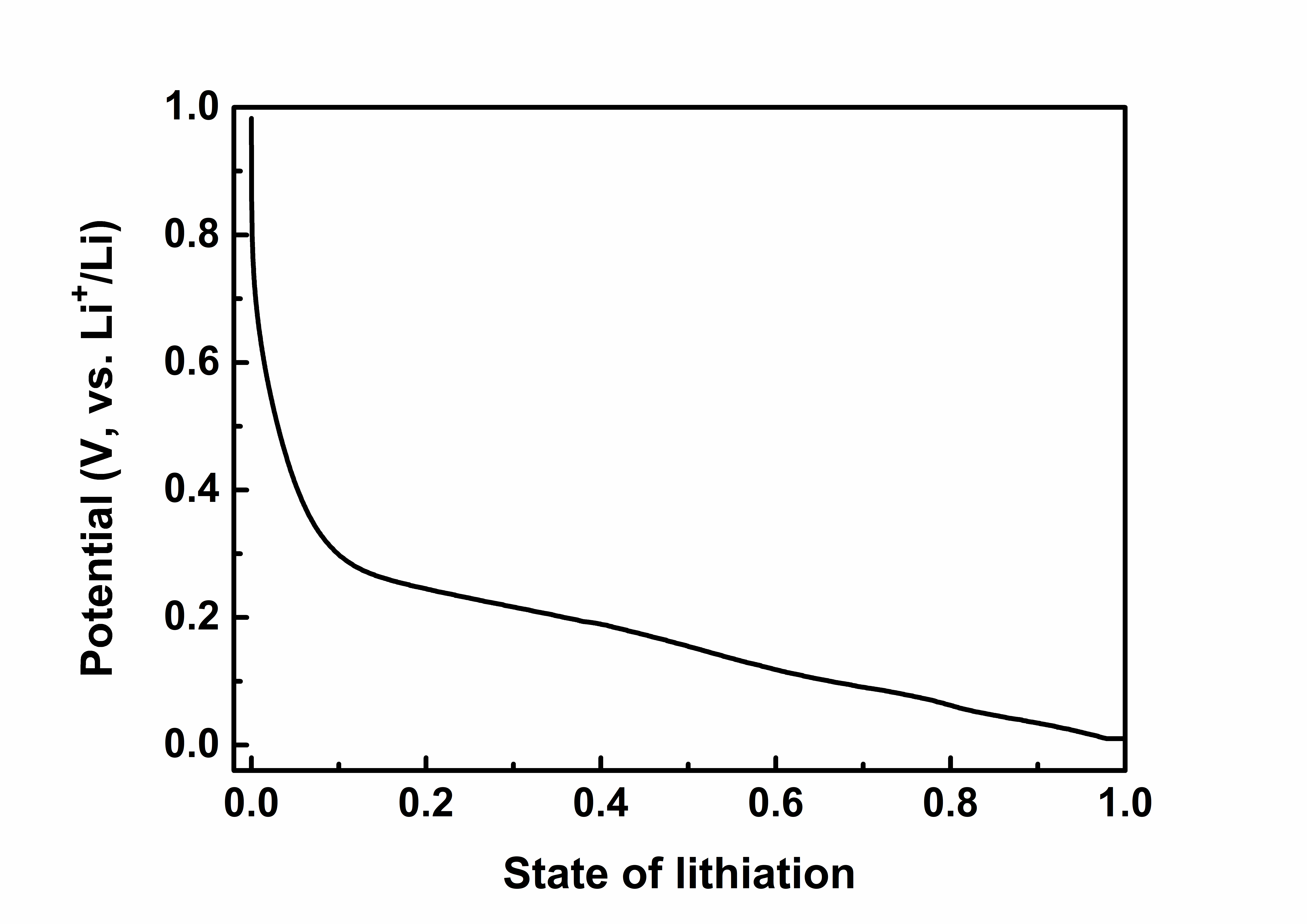


**Fig. S17.** Equilibrium potential of Li in SiO@C composites as a function of the state of lithiation ([Li^+^]/[Li^+^]_max_).


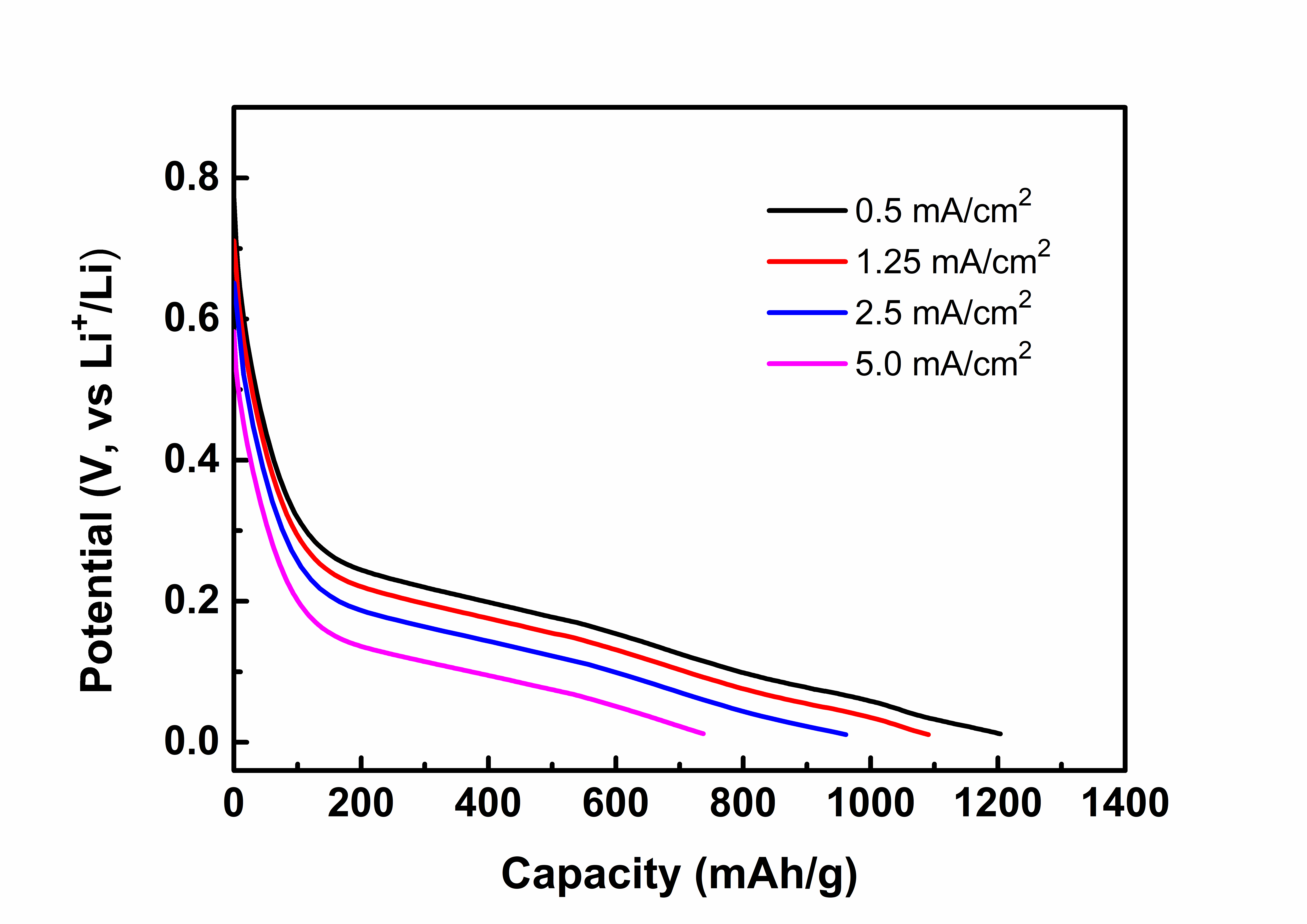


**Fig. S18.** Simulation of lithiation profiles of SiO@C-l with different rate.


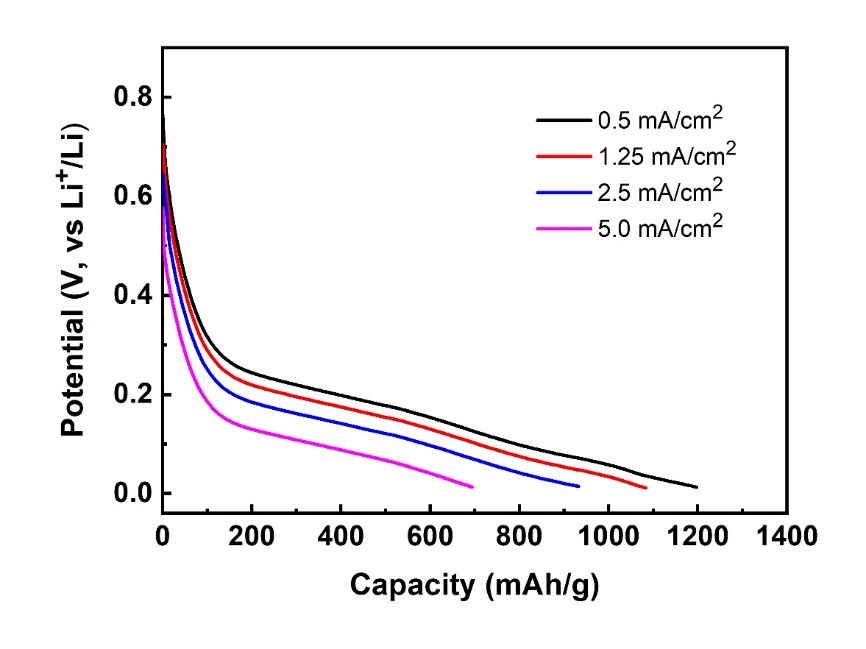


**Fig. S19.** Simulation of lithiation profiles of SiO@C-m with different rate.


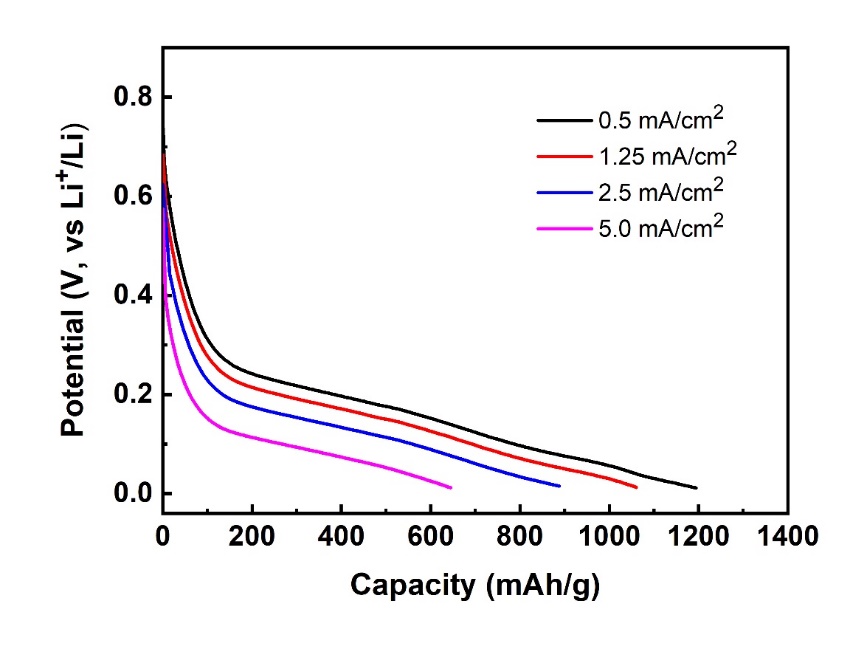


**Fig. S20.** Simulation of lithiation profiles of SiO@C-h with different rate.


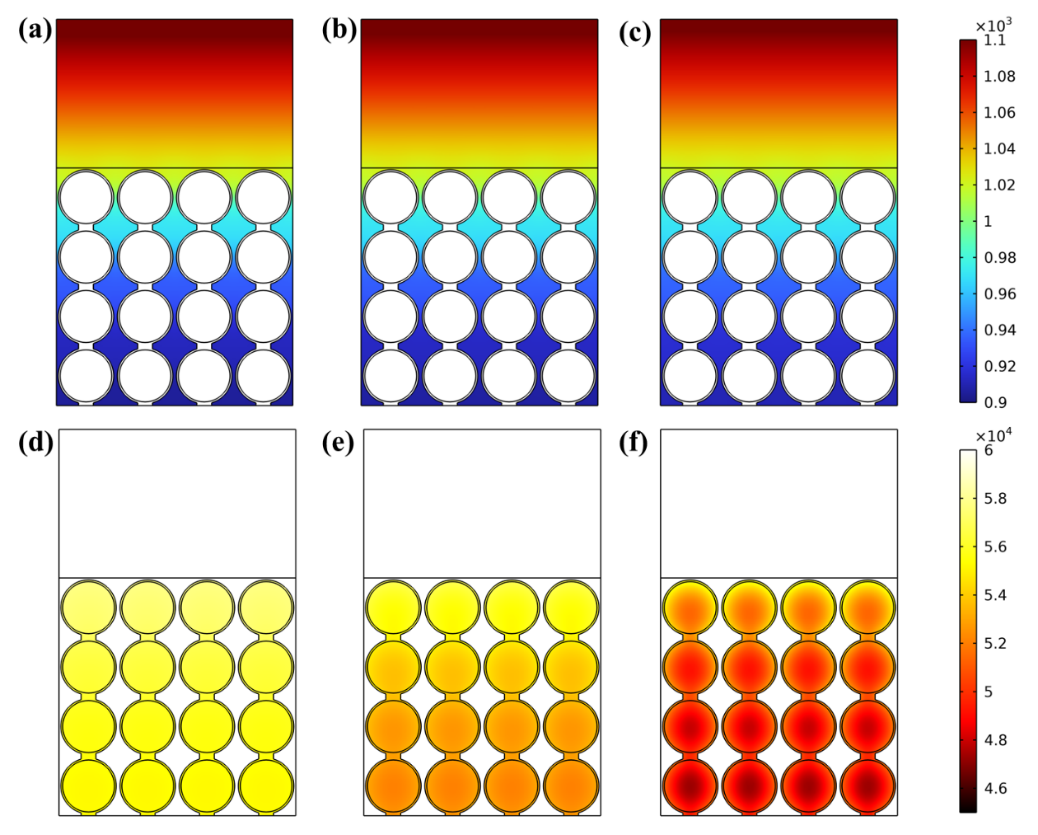


**Fig. S21.** Distribution of the Li^+^ concentration in the electrolyte (a-c) and in solid phase (d-f) at the end of discharge for (a)(d) SiO@C-l, (b)(e) SiO@C-m and (c)(f) SiO@C-h.


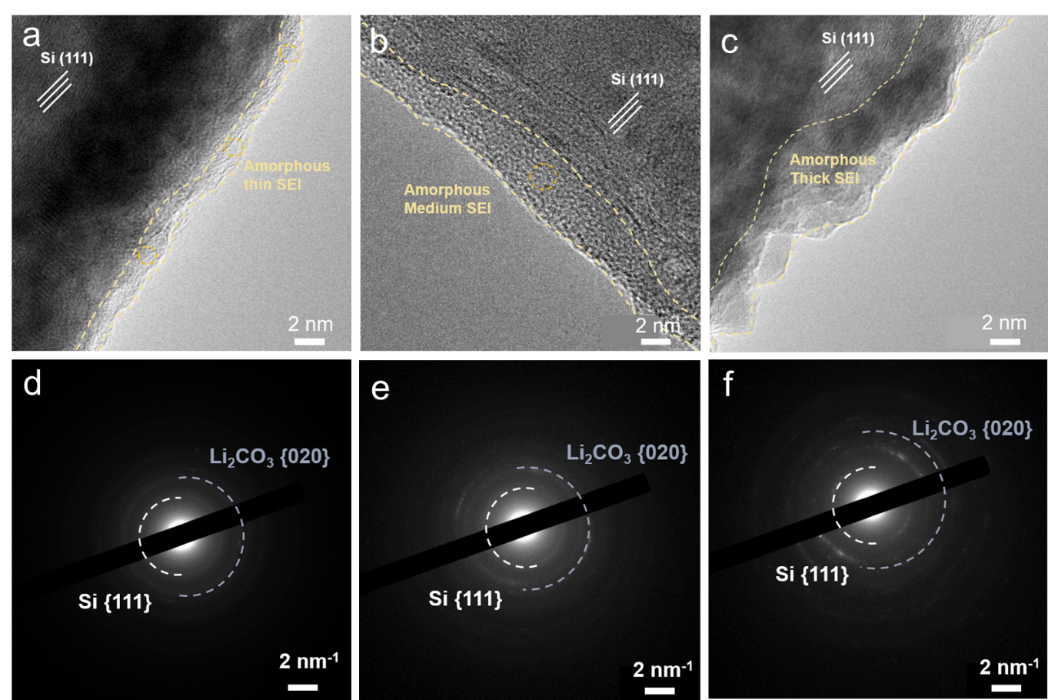


**Figure S22.** The delithiated Cryo-TEM and corresponding selected area electro diffraction pattern of different SiO@C composites. (a) (d)SiO@C-l; (b) (e)SiO@C-m; (c) (f)SiO@C-h.


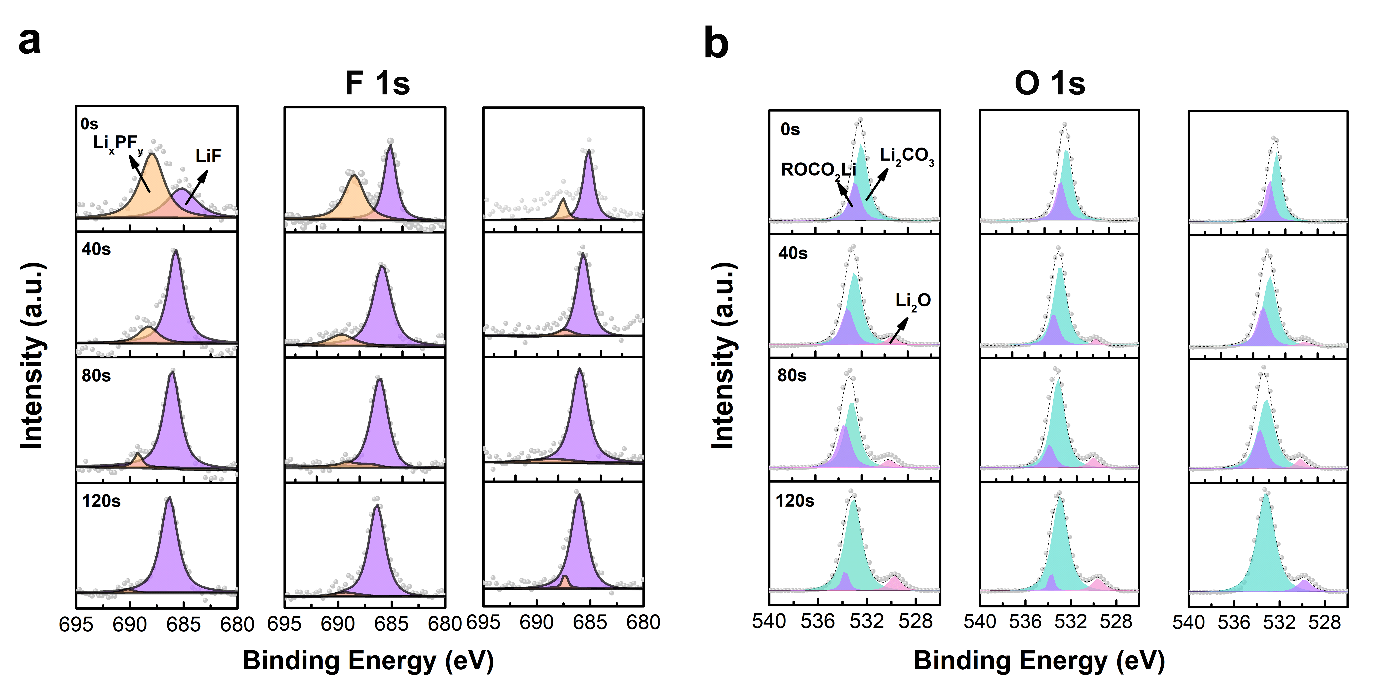


**Figure S23.** High-resolution XPS spectra of (a) F 1s, (b) O 1s for different alkali solubility composite (𝛼 decreases from left to right).


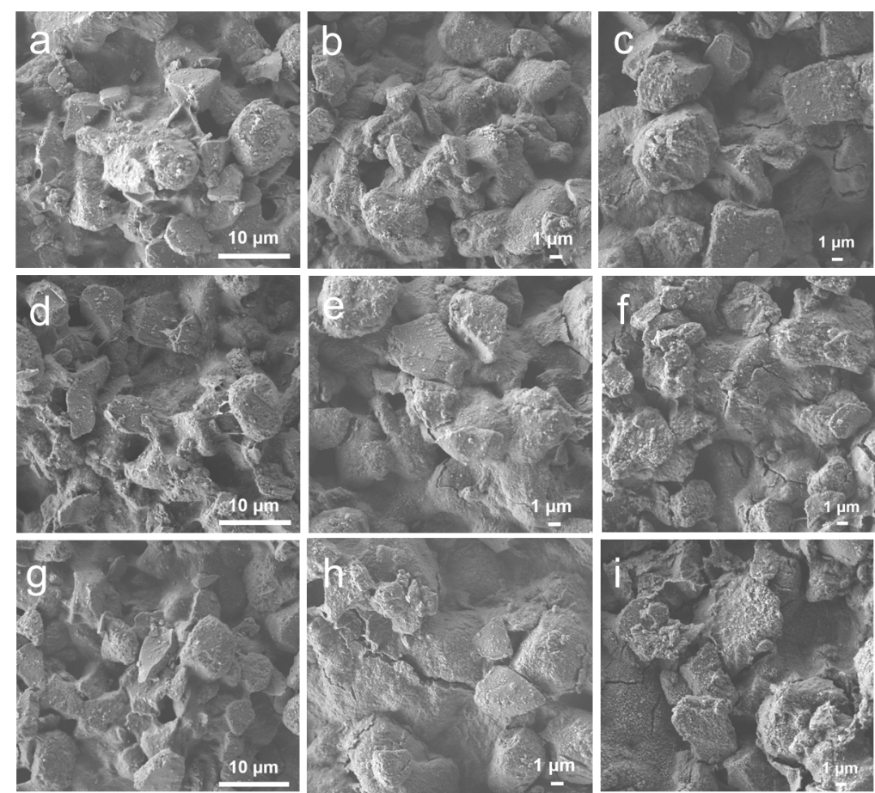


**Figure S24.** Top-view SEM images of electrode surface for different SiO@C composites under different cycling condition. (a-c) SiO@C-l (d-e) SiO@C-m (g-i) SiO@C-h correspond pristine electrode, after 1^st^ cycling and after several rate cycling, respectively.





**Figure S25.** XRD of SiO@C composites with different conductive network after cycling.





**Figure S26.** Raman spectra of SiO@C composites with different conductive network after cycling.


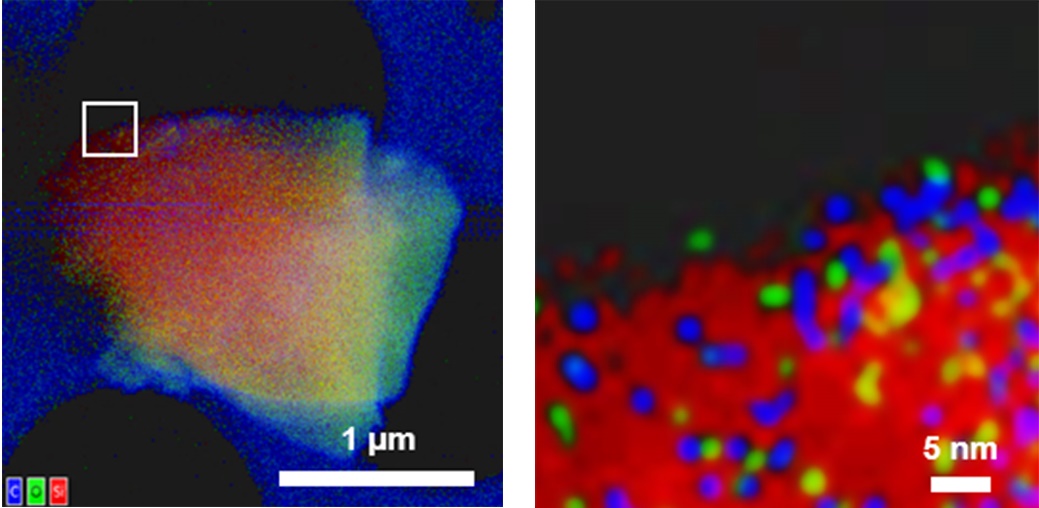


**Figure S27.** EDS images with low and high magnification times of SiO@C-h.


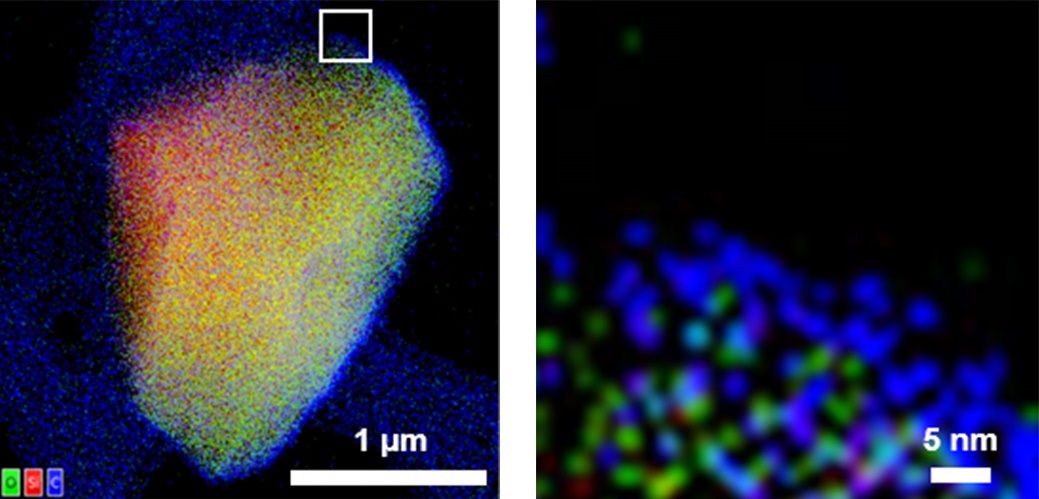


**Figure S28.** EDS images with low and high magnification times of SiO@C-m.


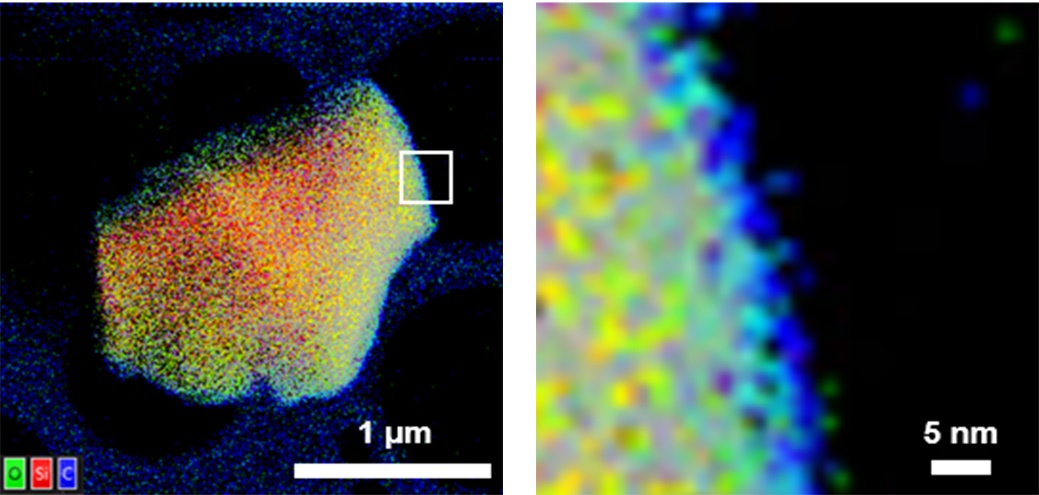


**Figure S29.** EDS images with low and high magnification times of SiO@C-l.

**
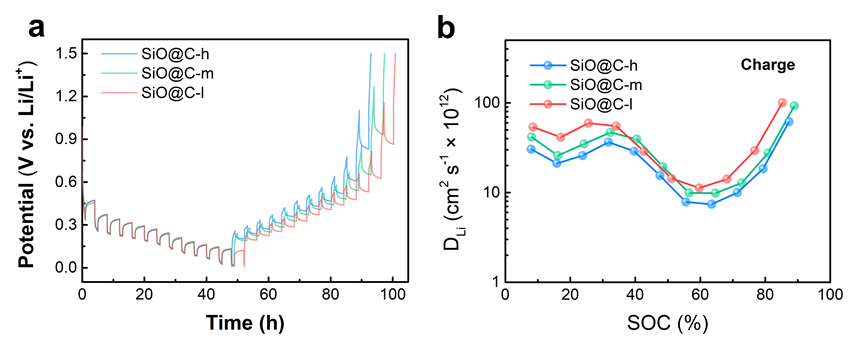
**

**Figure S30.** GITT profiles and Li^+^ diffusion rate in different SOC of SiO@C-h, SiO@C-m, SiO@C-l composites during the 2^nd^ cycle.

GITT-derived apparent lithium diffusion coefficient (D_Li_^+^) for SiO-based composites was calculated according to the below equation [1, 2, 3, 4]:

$$D_{^{^{Li^{+}}}}=\frac{4}{\pi\tau}\left(\frac{m_{B}V_{M}}{M_{B}S}\right)^{2}(\frac{\Delta E_{S}}{\Delta E_{t}})^{2}$$

where *m_B_* and *M_B_* are the active and molar masses, respectively, *V_M_* is the molar volume, *S* is the active surface area of electrode, and *τ*, Δ*E_S_*, and Δ*E_τ_* are respectively the pulse time, voltage change between steps, and voltage change during the pulse period.

**

**

**Figure S31.** Rate performance of SiO@C-h, SiO@C-m, SiO@C-l composites at 6 A g^-1^ and 8 A g^-1^.

**Model description and parameters**

**Table S1.** Governing equations and definite conditions of the 2D electrochemical model

| **Lithium foil electrode** | |
| --- | --- |
| Current on the surface | $\mathop{i_{loc,Li}}\limits^{.} =i_{Li}^{0}\left[\exp(\frac{\alpha_{a,Li}F\eta}{RT})-\exp(\frac{-\alpha_{c,Li}F\eta}{RT})\right]$ $i_{Li}^{0}=i_{\text{ref,Li}}^{0}\left(\frac{c_{1}}{c_{1, \text{ref}}}\right)^{\alpha_{a}}$     |
| Boundary conditions | $\mathbf{n}\cdot\mathbf{J}_{l}=-\frac{i_{loc,Li}}{F}$   |
| **SiO@C electrode** | |
| Solid phase diffusion | $\frac{\partial c_{s}}{\partial t}+\nabla\cdot\mathbf{J}_{s}=0$ $\mathbf{J}_{s}=-D_{s}\nabla c_{s}$   |
| Li-ion insertion kinetics | $\mathop{i_{loc,AM}}\limits^{.} =i_{AM}^{0}\left[\exp(\frac{\alpha_{a,AM}F\eta}{RT})-\exp(\frac{-\alpha_{c,AM}F\eta}{RT})\right]$ $i_{\text{AM}}^{0}=i_{\text{ref},\text{AM}}^{0}\left(\frac{c}{c_{s,\text{ ref }}}\right)^{\alpha_{c}}\left(\frac{c_{s,\max}-c}{c_{s,\text{ max }}-c_{s,\text{ ref }}}\right)^{\alpha_{a}}\left(\frac{c_{l}}{c_{1,\text{ ref }}}\right)^{\alpha_{a}}$     |
| Charge balance |  |
| Boundary conditions | $\mathbf{n}\cdot\mathbf{J}_{l}=\mathbf{n}\cdot\mathbf{J}_{s}=-\frac{i_{loc,AM}}{F}$ (interface of AM) $-\mathbf{n}\cdot\mathbf{i}_{s}=i_{\text{app}}$  (current collector) |
| Initial conditions | $c_{s}=0$ (inside of AM) |
| **Electrolyte** | |
| Mass transport in electrolyte | $\mathbf{J}_{l}=-D_{l,\text{eff}}\nabla c_{l}+\frac{\mathbf{i}_{l}}{F}$ $\nabla\cdot\mathbf{J}_{l}=0$ $D_{l,\text{eff}}=\varepsilon_{l}^{1.5}D_{l}, \sigma_{l,\text{eff}}=\varepsilon_{l}^{1.5}\sigma_{l}$       |
| Charge balance | $\nabla\cdot\mathbf{i}_{l}=0$ |
| Boundary conditions | $\mathbf{n}\cdot\mathbf{J}_{l}=\mathbf{n}\cdot\mathbf{J}_{s}=-\frac{i_{loc,AM}}{F}$ |
| Initial conditions | $c_{l}=c_{0}$ |

**Table S2.** Parameters of the 2D electrochemical model

| **Parameter** | **Symbol** | **Value/Definition** |
| --- | --- | --- |
| Anodic charge transfer coefficient of SiO@C | $\alpha_{a,\text{AM}}$ | 0.5 |
| Cathodic charge transfer coefficient of SiO@C | $\alpha_{c,\text{AM}}$ | 0.5 |
| Anodic charge transfer coefficient of Li foil | $\alpha_{a,\text{Li}}$ | 0.5 |
| Cathodic charge transfer coefficient of Li foil | $\alpha_{a,\text{Li}}$ | 0.5 |
| Porosity of the separator | $\varepsilon_{l}$ | 0.4 |
| Electronic conductivity of AM | $\sigma_{s}$ | Measured |
| Ionic conductivity of the electrolyte | $\sigma_{l}$ | 0.95 S/m |
| Standard concentration in the electrolyte | $c_{0}$ | 1000 mol/m^3^ |
| Li concentration in the AM | $c_{s}$ | - |
| Maximal Li concentration in AM | $c_{s,\max}$ | 1e5 mol/m^3^ |
| Diffusion coefficient of Li^+^ in the electrolyte | $D_{l}$ | 3.8e-10 m^2^/s |
| Li diffusion coefficient in AM | $D_{s}$ | Measured |
| Faraday constant | $F$ | 96485 C/mol |
| Exchange current density of SiO@C | $i_{\text{ref,AM}}^{0}$ | Measured |
| Exchange current density of Li | $i_{\text{ref,Li}}^{0}$ | 10 A/m^2^ |
| Li transference number | $t_{+}$ | 0.251 |
| Equilibrium potential of Li | $E_{\text{eq,Li}}$ | 0 V |
| Equilibrium potential of AM | $E_{\text{eq,AM}}$ | Show as Fig. S17 |

**Table S3.** Electrical conductivity of the three SiO@C samples without pressure

| Samples | Conductivity/S·cm^-1^ |
| --- | --- |
| SiO@C-l | 0.136 |
| SiO@C-m | 0.120 |
| SiO@C-h | 0.112 |

References

1. Z. Zhao, J. Han, F. Chen, J. Xiao, Y. Zhao, Y. Zhang, D. Kong, Z. Weng, S. Wu, Q.-H. Yang. Liquid metal remedies silicon microparticulates toward highly stable and superior volumetric lithium storage. Adv. Energy Mater. **12**(7), 2103565 (2022). <https://doi.org/https://doi.org/10.1002/aenm.202103565>

2. X. Wan, C. Kang, T. Mu, J. Zhu, P. Zuo, C. Du, G. Yin. A multilevel buffered binder network for high-performance silicon anodes. ACS Energy Lett. 3572-3580 (2022). <https://doi.org/10.1021/acsenergylett.2c02030>

3. Y. Huang, B. Shao, Y. Wang, F. Han. Solid-state silicon anode with extremely high initial coulombic efficiency. Energy Environ Sci. (2023). <https://doi.org/10.1039/D2EE04057C>

4. S. Kim, D.-Y. Han, G. Song, J. Lee, T. Park, S. Park. Resilient binder network with enhanced ionic conductivity for high-areal-capacity si-based anodes in lithium-ion batteries. Chem. Eng. J. **473**(145441 (2023). <https://doi.org/https://doi.org/10.1016/j.cej.2023.145441>
